# Bacteriobiota of the Cave Church of Sts. Peter and Paul in Serbia—Culturable and Non-Culturable Communities’ Assessment in the Bioconservation Potential of a Peculiar Fresco Painting

**DOI:** 10.3390/ijms24021016

**Published:** 2023-01-05

**Authors:** Ivica Dimkić, Milica Ćopić, Marija Petrović, Miloš Stupar, Željko Savković, Aleksandar Knežević, Gordana Subakov Simić, Milica Ljaljević Grbić, Nikola Unković

**Affiliations:** Faculty of Biology, University of Belgrade, Studentski trg 16, 11 000 Belgrade, Serbia

**Keywords:** bacteriobiota, microbial communities, bioconservation, biological control, whole-genome sequencing, wall painting

## Abstract

The principal aim of this study was to determine bacterial diversity within the Cave Church of Sts. Peter and Paul, via culturable and non-culturable approaches, and elucidate the antifungal potential of autochthonous antagonistic bacterial isolates against biodeteriogenic fungi. Furthermore, whole-genome sequencing of selected bacterial antagonists and the analysis of genes included in the synthesis of secondary metabolites were performed. With the highest RA values, determined in metabarcoding analysis, phyla *Actinobacteriota* (12.08–54.00%) and *Proteobacteria* (25.34–44.97%) dominated most of the samples. A total of 44 different species, out of 96 obtained isolates, were determined as part of the culturable bacteriobiota, with the predominance of species from the genus *Bacillus*. *Bacillus simplex* was the only isolated species simultaneously present in all investigated substrata within the church. The best antagonistic activity against 10 biodeteriogenic fungi was documented for *Streptomyces anulatus*, followed by *Bacillus altitudinis*, *Chryseobacterium viscerum*, and *Streptomyces* sp. with their highest PGI% values ranging of from 55.9% to 80.9%. These promising results indicate that characterized bacteria are excellent candidates for developing biocontrol strategies for suppressing deteriogenic fungi responsible for the deterioration of investigated fresco painting. Finally, isolate 11-11MM, characterized as *Streptomyces* sp., represents a new species for science prompting the need for further study.

## 1. Introduction

The Cave Church of Sts. Peter and Paul was built on Stara Planina Mountain, 22 km from the town of Pirot in the vicinity of the village Rsovci, the Republic of Serbia, in a limestone cave on Kalik hill, and is well known for its fresco painting of the “Bald-headed Jesus”. The fresco is also known by its other name, “the young Jesus” or “Jesus the Infant”. It is the only fresco painting present in the church and was painted on its northern wall. Serbian art historians emphasize that among fresco painters there are those who believe that the painting of the “Bald-headed Jesus” is the work of artists influenced by various denominations of Christianity, while others believe it to be the work of hermits from Sinai, who painted it during their stay in the cave on one of their pilgrimages. No accurate data exists on when it was painted. Based on the style of painting and the pigments used, it dates from the middle or second half of the 13th century, while others believe it belongs to a much older school of painting. At the time, hermit cells were not subject to church canon laws and rules on how to represent saints. Therefore, it is assumed that Jesus was painted in a manner that indicates that the painters were unaffected by the conventional painting techniques of that era, and accordingly, it is assumed that the painting very likely predates the 13th century. This fresco, unique among Orthodox fresco paintings, depicts Jesus as very young, bald-headed, dressed in Buddhist robes, with a divine halo, and an octagonal star, while three of his fingers are raised, signifying baptism [1]. Why Jesus was depicted in such a manner is still a mystery. It is precisely because of these reasons that the fresco painting and the entire cave church have been under the protection of the state since 1981 and have been a part of the cultural property of Serbia since 1981 [2].

If we accept the fact that cultural property is the best witness to the culture and history of a nation, then the responsibility of its preservation falls to the state and its institutions. They do so by relying on legal regulations, but also by relying on a series of measures such as conservation, restoration, and reconstruction. The destruction of cultural heritage and buildings of historical importance is the result of both natural and anthropogenic factors. Unfortunately, as a result of neglect, cultural property is left exposed to negative environmental impacts and changes, the most relevant of which are those resulting from the activity of microorganisms, which leads to biodeterioration. As a process, biodeterioration is the result of manifold activities of microalgae, bacteria, cyanobacteria, and fungi including lichens, which form complex communities, and by means of their ability to colonize various types of substrates, cause the destruction of objects of historical value that are a part of a nation’s cultural heritage (paper, parchment, oil paintings, wood, stone monuments, frescoes, etc.) [3]. Environmental parameters and factors such as humidity, increased temperature, pH value, and light have an unfavorable impact on cultural properties, but at the same time, they create favorable conditions for the growth of deteriogenic microorganisms. They in turn lead to the destruction of objects by means of causing biocorrosion, changes to color, creation of crusts, exfoliation of paint layers and mechanical degradation [4]. Through their metabolic activities, they damage the appearance of cultural property and, at the same time, consume the organic nutrients within the material and pigments found within its colors [5].

Over the past few decades, churches and monasteries, as well as their fresco paintings, have become the focus of microbiological studies, with special attention being devoted to their painted parts [6]. Fresco painting, or painting on stone materials, is a traditional technique of painting, whereby the pigment is applied directly to a wet lime plaster surface. Considering that throughout history, artists have used natural materials in the painting process, including natural oils to bind the pigments, natural inorganic (obtained from digging in the ground) and organic (obtained from plant or animal wildlife) pigments, egg yolk rich in protein and fatty acids, as well as animal fat, this created an ideal substrate for microorganisms which possessed the ability to decompose the natural components of paintings [7]. Microstratigraphic analyses determined that lime plaster was mixed with various types of aggregates such as quartz, feldspar, amphibole, and volcanic rock fragments and that calcium carbonate was also used to bind pigment. Fourier-transform infrared and micro-Raman spectrometry analyses are widely used in the study of the components of pigments in frescoes the world over, and one such study revealed that the color yellow required the use of litharge, that calcium carbonate was used to make white, soot to make black, and hematite and iron silicate to make shades of red [8]. The natural surface porousness of frescoes makes them receptive to microbial spores and to airborne cells [9]. By colonizing such surfaces, microorganisms destroy the material which lies at its base, impacting the physical, chemical, and bioreceptive properties of the material and the substrate. The pioneering microorganisms can then be enabled to create specific biofilms via colonization, which in turn can alter the thermal properties and moisture content of the material, and consequently impact the activities of other microorganisms, which in turn leads to alteration in original coloration, porousness, and moisturizing of the substrate [10]. The susceptibility of stone to the colonization of organisms represents its bioreceptivity, a term introduced by Guillitte, back in 1985. He divided bioreceptivity into primary (which indicates the possibility of healthy material being colonized by organisms), secondary (which is the result of abiotic and biotic factors), and tertiary (which is initiated by the number of nutrients in the substrate) [11]. Bioreceptivity is affected by the porousness of the material, due to its ability to retain excess water, as well as by the roughness of the surface which impacts surface particle accumulation. Any kind of material protection shielding it from being dried out (from the sun’s rays and wind), and from other abiotic factors also impacts bioreceptivity..

Most studies have focused on the destruction of the material of paintings and frescoes caused by the colonization of microbes [12]; while very few have focused on the biological decay of paintings. The processes of conservation and restoration have special significance but are also predetermined by a good knowledge of the structure of the population of microorganisms colonizing cultural heritage objects. Cyanobacteria, algae, and lichens are pioneer colonizers which appear before the active development of heterotrophic bacteria and other fungi [13]. Numerous studies determined that the inner walls of locations with a constant water supply and no direct sunlight are dominated by algae and cyanobacteria. These locations undergo changes in the intensity of the color green, depending on the level of development of the communities, while other colors, such as pink or yellow, indicate the production of pigment by *Actinobacteria* and certain algae [14]. The microbiota found on cultural heritage objects undoubtedly represents an ecosystem consisting of various groups of microorganisms whose activity leads to destruction. Methods such as the mechanical, physical, and chemical removal of their deposits from surfaces are most commonly used but have not proven to be a long-term viable solution. The proper identification of these organisms, as well as the preparation of an adequate strategy for the mitigation of their impact, is challenging, but nevertheless, it is a necessary step in the successful control of the biodegradation of cultural heritage objects. Earlier studies focused on cultivable microorganisms, but without focusing on their function in the biodeterioration process. The most frequently detected bacteria in wall paintings or paintings on similar substrates were from the *Bacillus*, *Pseudomonas*, *Micrococcus*, *Staphylococcus*, *Paenibacillus*, *Nocardia*, and *Streptomyces* genera [15]. This was the practice prior to the inclusion of the PCR method in microbiological studies and the detection of microbial communities independently of cultivation and their isolation [6]. Today, metabarcoding studies and next-generation sequencing provide more relevant results and descriptions of non-culturable communities of microorganisms [3]. Most recent studies involve research on the diversity and distribution of microorganisms on different cultural heritage objects and deal with the stone substrata and masonry including stone monuments [16], cathedral and church walls [17,18], as well as buildings for different purposes [19]. The mentioned studies deal with both bacterial and fungal communities. A recent study by Dziurzynski et al. (2020) uses metabarcoding analyses even for the assessment of the bacterial community of the museum’s indoor air [20].

The application of biotechnology in cultural heritage conservation and preservation has, over the last two decades, provided a successful new alternative to the traditional approach and is gaining more traction in today’s climate of developing novel environmentally friendly and safe procedures. The main idea lies in the fact that only a few microorganisms deteriogenic in the natural processes, while the majority of them are responsible for “virtuous” processes [21]. With that goal in mind, the new paradigm focuses on GRAS group of beneficial bacteria (Risk group 1) and their metabolites as a new tool for elimination of fungal infestation and safeguarding of artworks with great advantages in terms of safety, effectiveness, costs, and environmental sustainability. Bacteria of Bacillus genus are of special importance in that regard since they synthesize a variety of bioactive molecules, e.g., ribosomally and non-ribosomally synthesized antimicrobial peptides, with potent antagonistic activities against biodeteriogenic fungi [22,23,24]. Furthermore, hypogean environments represent robust microbial cell factories with unexplored biosynthetic potential of strains that potentially could be applied for the development of a future generation of biocides and safe sustainable methods 22].

To the best of our up-to-date knowledge, the literature on culturable and total bacteriobiota of unique geoheritage sites such as rare cave churches is non-existent, although studies of the bacterial diversity within caves have been carried out in the past, some of which host very valuable Paleolithic paintings [25,26,27,28]. As a part of the broader research project, aimed at determining the main causes of decay of numerous cultural heritage objects in Serbia and developing alternative, non-invasive and safe biocontrol formulation applicable during conservation treatments, the principle purpose of this research phase was to determine bacterial diversity, via culturable and non-culturable approaches, of the unique fresco painting, iconostasis, and limestone walls within the rare subterranean habitat of Cave Church of Sts. Peter and Paul. The isolation of autochthonous antagonistic bacteria and evaluation of the antifungal potential of selected bacterial isolates against the biodeteriogenic fungi previously isolated from the same origin and responsible for some of documented alterations [29], together with the whole-genome sequencing of the most promising bacterial antagonists and the analysis of genes included in the synthesis of secondary metabolites, were accomplished as the necessary first step in the development of novel bioformulation whose potential application in situ would achieve the desired effect of sustainable and long-term suppression of fungal infestation of studied works of art.

## 2. Results

### 2.1. Diversity of the Cave Church of Sts. Peter and Paul Microbiome

A total of 1,737,757 raw sequences were obtained from the 16S libraries sequencing (from 94,529 to 223,434 per sample). After primer removal, denoising, quality filtering, length trims, and sequence decontamination, the number of reads ranged from 50,099 up to 156,829 (Table S1). The mean length of obtained ASV sequences was 415. Based on alpha rarefaction curves (Figure S1), since there were evident differences in reads among samples, diversity indices were estimated after rarefaction to even depth according to the sample with the lowest number of reads (sample 06).

#### 2.1.1. The Analysis of Alpha and Beta Diversities 

The initial study of the diversity and richness of bacterial communities was carried out in relation to the different origins of the sampled material (samples were divided into samples taken from the fresco and around the fresco) on four taxonomic levels (the phylum, family, genus, and the ASV), as presented in Table 1. 

The indices which denote the richness of bacterial species (OBS, Chao1 and ACE) showed that compared to all the studied taxonomic levels, the highest richness of species was noted for the samples taken around the fresco. As far as indices that indicate bacterial diversity are concerned (the Shannon and Gini–Simpson index), the ASV Shannon index indicated a simultaneously high level of bacterial diversity on the fresco and around it. At the genus and the family taxonomic levels, a relatively good diversity was noted, while at the phylum level there was no difference, and the lowest level of diversity was noted. However, statistically significant differences, for all indices of the fresco and around it, according to the Kruskal–Wallis test were not observed at all taxa levels (*P* < 0.05). 

Reviewing the samples individually, it was noticed the highest bacterial richness at the AVS taxa level originated from samples 04–05, taken from the fresco surface damage area, while the same richness is obtained for samples originating from the cave interior wall deposits (in increasing order, 07 > 09 > 11 > 08) (Table S2). Samples 01, 02, and 03, collected from the fresco itself, also showed high richness and diversity according to both indices, while the lowest values were noted for the iconostasis. Sample 10, obtained from the white deposits, showed, among all samples from the wall deposit, the lowest richness and diversity at all taxa levels.

**Figure 1:**
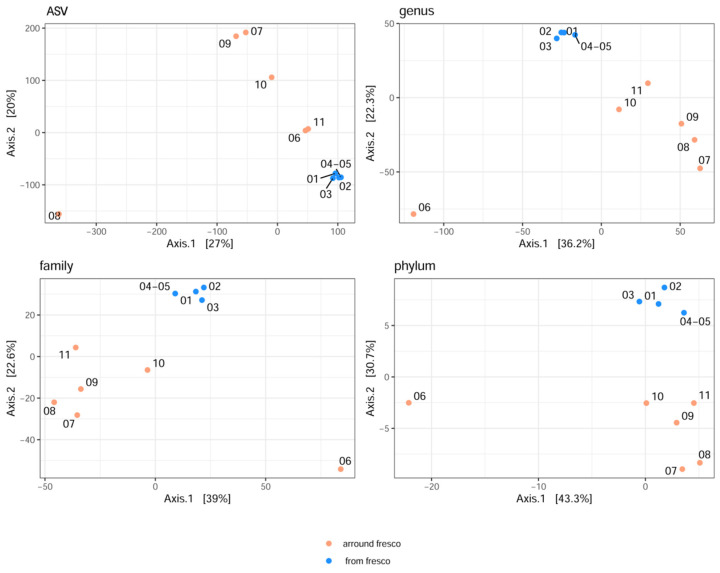
The MDS (PCoA) plot was created with Euclidean distances at the ASV, genus, family, and phylum taxa levels, showing the variability between samples.

For visualization of beta diversity, the initial analysis was performed with Bray–Curtis dissimilarities, and samples were rarefied to even depth (variability between samples as DPCoA plot is shown in Figure S2). Based on the results, it can be concluded that the samples from the fresco are jointly together. The majority of side wall samples showed that the furthermost distance (at all taxonomic levels) was obtained for the samples, which originated from the fresco environment (samples 07 and 08 and samples 09 and 11 were grouped). Moreover, samples 06 and 10 were closely related to the samples from the fresco. However, a more suitable stabilization of variance was carried out later, and results were obtained by multidimensional scaling (MDS or PCoA) based on the Euclidean distance (Figure 1).

Values on the axes were scaled based on their own values. The results obtained indicate that in terms of microbiome diversity on all the taxonomic levels (explaining 27%, 36.2%, 39%, and 43.3% of variability, respectively), the samples from the fresco itself were similar to one another, as were the samples obtained from the side walls of the church. The greatest diversity at the level of the phylum, family, and genus, was noted for the sample obtained from the iconostasis (sample 06), while at ASV level, sample 08 was considerably distanced from the rest of them. However, at ASV level, samples 06 and 11 were closer in composition to the samples obtained from the fresco.

**Figure 2:**
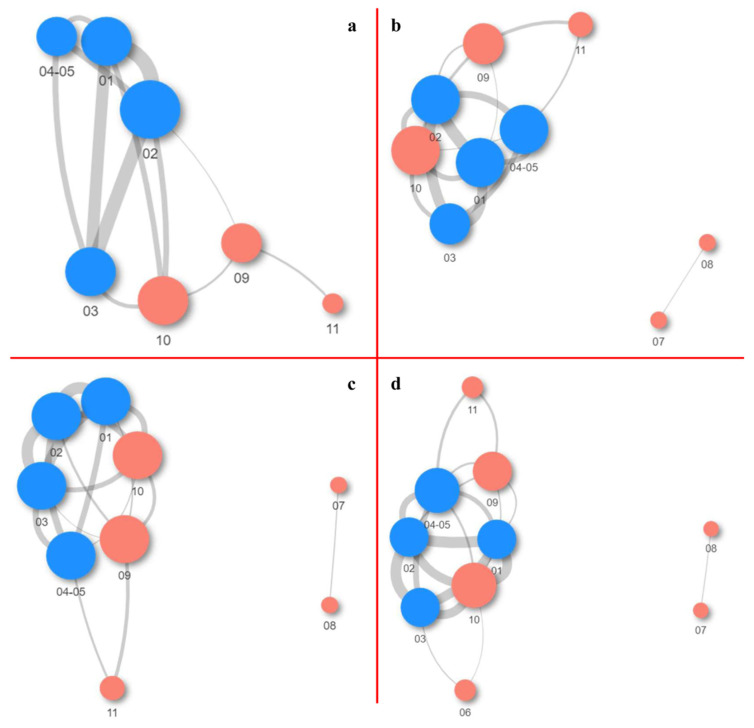
Network analysis of samples with Weighted UniFrac distances at the: (**a**) ASV; (**b**) genus; (**c**) family; and (**d**) phylum taxa levels. Node size corresponds to the number of connections, while edge width corresponds to the distance among samples (closer distances equal wider edges). Red nodes are from the samples around the fresco, while blue nodes are the fresco’s samples.

In networking analysis, weighted UniFrac distances were used to visualize the distances among samples, which took into consideration the phylogenetic distances between the obtained taxa (Figure 2). Based on the results obtained on all the taxonomic levels, with the exception of ASV, samples 07 and 08 were considerably more distant from the other samples and possessed only one link which was, however, at a suitable mutual distance. Sample 02, at the ASV level, had the greatest number of connections with other samples (the number of links was 5), and it was, according to the established proximity, closest to sample 01. At the genus level, the situation was somewhat more complex, whereby samples 01, 02, 04-05, and 10 were mutually connected with as many as 5 links. A closer distance was noted between the samples taken from the fresco, while sample 10, even though close, was further removed based on the thickness of its linkage which, on average, was approximately 0.07. Sample 06, even though considerably further, achieved two connections with samples 03 and 10 (only at the phylum level), while in the other cases, it was excluded from analysis due to its greater distance from the remaining samples. 

#### 2.1.2. A Taxonomic Analysis of the Total Bacteriobiota

**Figure 3:**
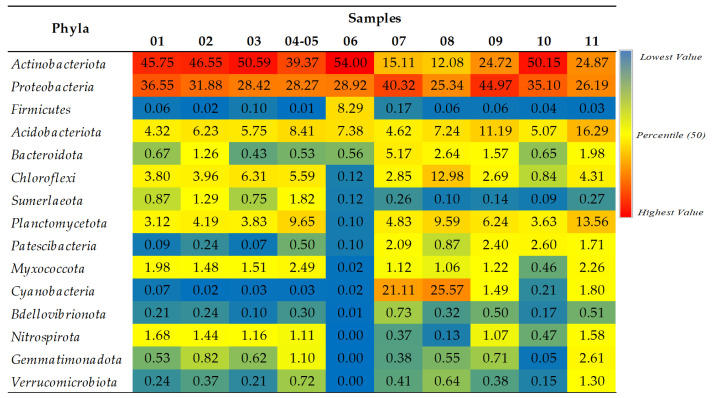
Relative abundance (RA) of bacterial phyla according to SILVA 138 database. Only taxa with a total percentage abundance above 0.5% for all samples were included. Blue, yellow, and red colors represent the lowest, medium, and highest RA, respectively.

The dominance of the phyla *Actinobacteriota* and *Proteobacteria* was noted for all the studied samples, except those obtained from the walls with blue-green deposits and patina with the greatest abundance of *Cyanobacteria* (samples 07 and 08) (Figure 3). The remaining phyla, such as *Acidobacteriota*, *Chloroflexi*, *Sumerlaeota*, *Planctomycetota*, and *Myxococcota*, were also found in higher percentages in the samples obtained from the fresco and the surrounding walls. The samples obtained from the wall deposits abounded in bacteria from the phyla *Bacteroidota*, *Patescibacteria*, and *Bdellovibrionota*, while *Nitrospirota* was evenly distributed among all the samples obtained from the fresco. On the iconostasis, in addition to *Actinobacteriota*, *Proteobacteria*, *Acidobacteriota*, and *Bacteridota*, the dominance of the phylum *Firmicutes* was also noted. The phyla with a considerably lower relative abundance (RA) were *Gemmatimonadota* and *Verrucomicrobiota*. 

**Figure 4:**
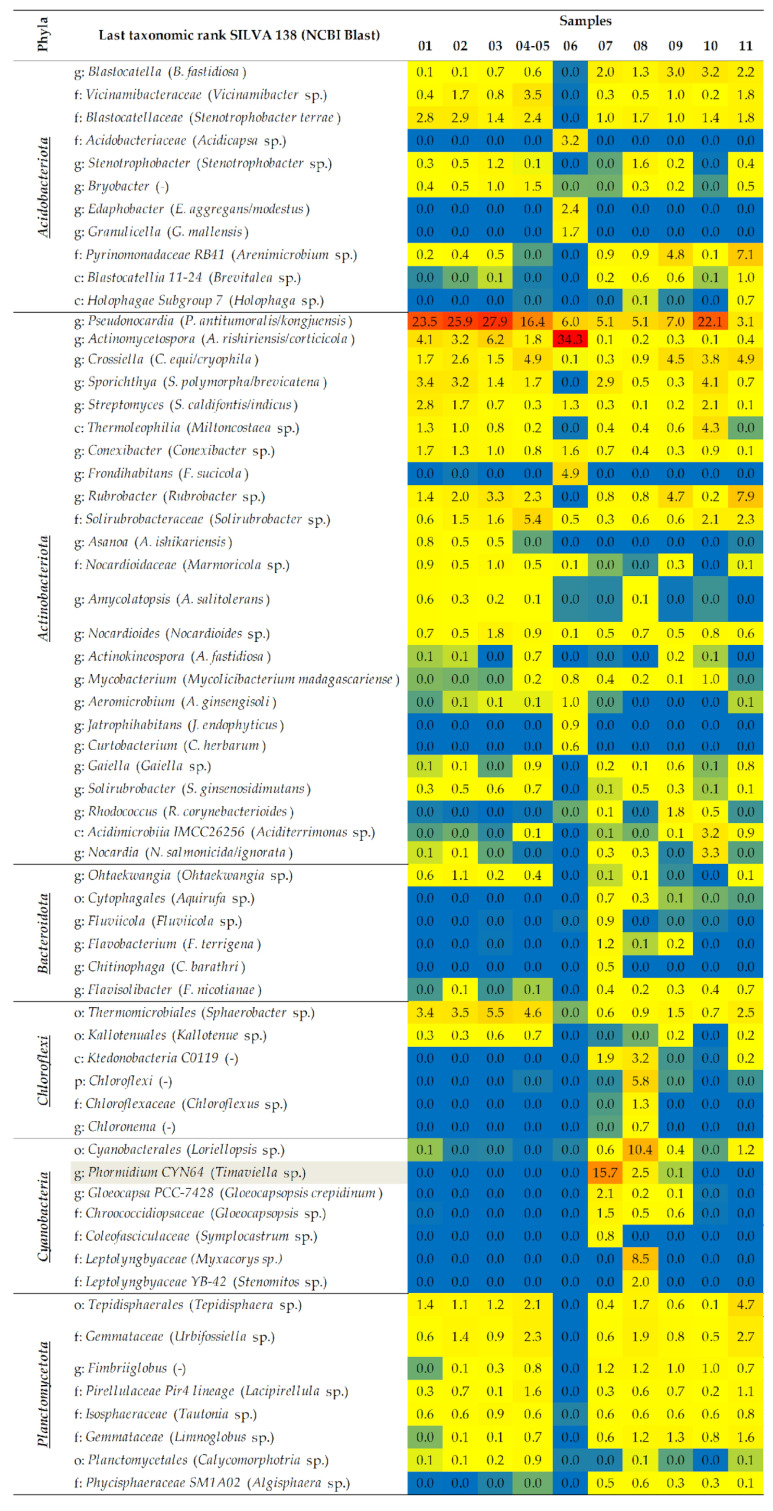

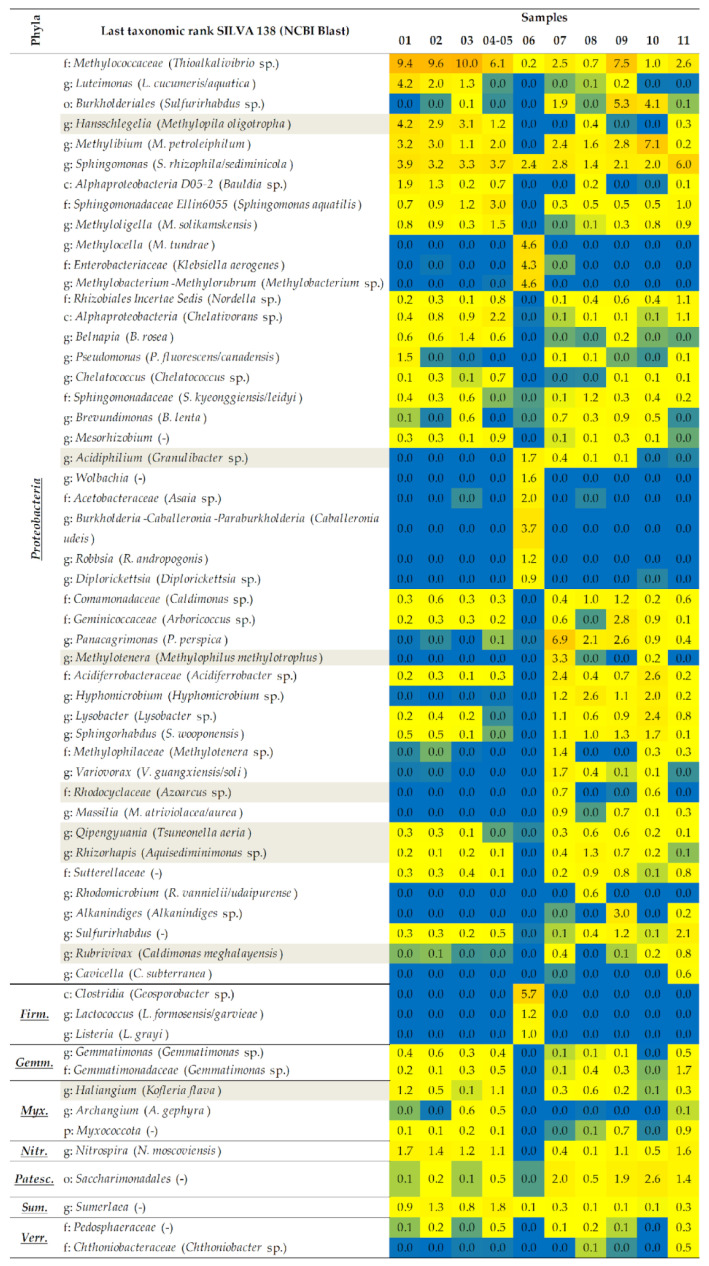
Relative abundance of bacterial taxa at the last taxonomic rank according to the SILVA 138 database and BLAST best top 10 hits in NCBI at the species level. Relative abundance of bacterial taxa are present at all proportion levels, and different colors indicate low or high abundance (blue, yellow, and red colors—lowest, medium, and highest RA, respectively). (p)—phylum; (c)—class; (o)—order; (f)—family; (g)—genus; (-)—not determined. The gray color indicates differences between SILVA 138 and NCBI results. Only taxa with a total percentage abundance above 0.5% for all samples were included. Firm—Firmicutes; Gemm—Gemmatimonadota; Myx—Myxococcota; Nitr—Nitrospirota; Patesc—Patescibacteria; Sum—Sumerlaeota; Verr—Verrucomicrobiota.

The bacterial taxa which dominated the fresco and which were found in a greater or lesser relative abundance (RA) belonged to the genera of the *Thermomicrobiales*, *Methylococcaceae*, *Blastocatellaceae*, and *Nocardioidaceae* families, as well as to the genera of *Pseudonocardia*, *Actinomycetospora*, *Streptomyces*, *Conexibacter*, *Hansschlegelia*, *Sphingomonas*, *Belnapia*, *Haliangium* and *Nitrospira*, which, based on the results of NCBI studies, might be associated with the following species: *Sphaerobacter* sp., *Thioalkalivibrio* sp., *Stenotrophobacter terrae*, *Marmoricola* sp., *Pseudonocardia antitumoralis*/*kongjuensis*, *Actinomycetospora rishiriensis*/*corticicola*, *Streptomyces caldifontis*/*indicus*, *Conexibacter* sp., *Methylopila oligotropha*, *Sphingomonas rhizophila*/*sediminicola*, *Belnapia rosea*, *Kofleria flava* and *Nitrospira moscoviensis* (Figure 4). After evaluating the RA of the species identified in the sample obtained from the fresco’s surface damage (sample 04–05), the dominance of *Alphaproteobacteria* (*Chelativorans* sp.), *Tepidisphaerales* (*Tepidisphaera* sp.), *Planctomycetales* (*Calycomorphotria* sp.), *Vicinamibacteraceae* (*Vicinamibacter* sp.), *Solirubrobacteraceae* (*Solirubrobacter* sp.), *Gemmataceae* (*Urbifossiella* sp., and *Limnoglobus* sp.), *Pirellulaceae Pir4 lineage* (*Lacipirellula* sp.), *Sphingomonadaceae Ellin 6055* (*Sphingomonas aquatilis*), *Bryobacter*, *Crossiella* (*C. equi/cryophila*), *Gaiella*, *Methyloligella* (*M. solikamskensis*), *Chelatococcus*, *Mesorhizobium*, and *Sumerlaea* was determined, as their abundance was greater than in the other samples obtained from the fresco. 

Contrary to that, in the sample obtained from the gray discoloration on fresco (sample 03), in addition to *Pseudonocardia*, *Actinomycetospora*, and *Sphaerobacter*, which had the greatest RA, the dominance of genera such as *Stenotrophobacter*, *Rubrobacter*, *Nocardioides*, *Belnapia* (*B. rosea*) and *Brevundimonas* (*B. lenta*) was also noted. The samples obtained from the fresco depiction of Jesus (sample 01) and the surrounding area (sample 02) were characterized by higher percentages of *Sporichthya* (*S. polymorpha*/*brevicatena*), *Streptomyces* (*S. caldifontis*/*indicus*), *Amycolatopsis* (*A. salitolerans*), *Ohtaekwangia*, *Luteimonas* (*L. cucumeris/aquatica*), *Bauldia* sp., and *Sphingorhabdus* (*S. wooponensis*), while *Pseudomonas* (*P. fluorescens/canadensis*) was found solely in sample 01.

The opposite was noted for the iconostasis’ sample (sample 06). The genera *Pseudonocardia* and *Actinomycetospora* were omnipresent, while the remaining taxa were unique for this sample, with an RA greater than 0.5% [*Acidobacteriaceae* (*Acidicapsa* sp.), *Edaphobacter* (*E. aggregans*/*modestus*), *Granulicella* (*G. mallensis*), *Frondihabitans* (*F. sucicola*), *Aeromicrobium* (*A. ginsengisoli*), *Jatrophihabitans* (*J. endophyticus*), *Curtobacterium* (*C. herbarum*), *Methylocella* (*M. tundrae*), *Enterobacteriaceae* (*Klebsiella aerogenes*), *Methylobacterium*-*Methylorubrum* (*Methylobacterium* sp.), *Acidiphilium* (*Granulibacter* sp.), *Wolbachia*, *Acetobacteraceae* (*Asaia* sp.), *Burkholderia*-*Caballeronia*-*Paraburkholderia* (*Caballeronia udeis*), *Robbsia* (*R. andropogonis*), *Diplorickettsia*, *Clostridia* (*Geosporobacter* sp.), *Lactococcus* (*L. formosensis/garvieae*), and *Listeria* (*L. grayi*)]. 

The most prevalent taxa linked to the various deposits on the walls (black, white, and pink—samples 09, 10, and 11, respectively) were *Blastocatella* (*B. fastidiosa*) and *Crossiella*. At the same time, for samples 09 and 10, *Burkholderiales* (*Sulfurirhabdus sp.*) was detected in a higher abundance. For samples 09 and 11, a greater dominance of *Pyrinomonadaceae RB41* (*Arenimicrobium* sp.), *Rubrobacter*, and *Nitrospira* was noted. The taxa *Rhodococcus* (*R. corynebacterioides*), *Geminicoccaceae* (*Arboricoccus* sp.), and *Alkanindiges* were more frequent in sample 09. Sample 10, obtained in the proximity of the fresco itself, contained an abundance of *Pseudonocardia*, *Sporichthya*, *Streptomyces*, *Thermoleophilia* (*Miltoncostaea* sp.), *Acidimicrobiia IMCC26256* (*Aciditerrimonas* sp.), *Nocardia* (*N. salmonicida*/*ignorata*), *Methylibium* (*M. petroleiphilum*), *Acidiferrobacteraceae* (*Acidiferrobacter* sp.), *Lysobacter*, and *Sphingorhabdus*. Sample 11, also obtained from the proximity of the fresco, was characterized by *Sphaerobacter* sp., *Tepidisphaera* sp., *Urbifossiella* sp., *Sphingomonas* (*S. rhizophila/sediminicola*), *Rhizobiales Incertae Sedis* (*Nordella* sp.), *Alphaproteobacteria* (*Chelativorans* sp.), *Sulfurirhabdus* and *Gemmatimonadaceae* (*Gemmatimonas* sp.). Contrary to that, in sample 07 (blue-green stone wall deposits below the fresco), communities of cyanobacteria such as *Phormidium CYN64* (*Timaviella* sp.), *Gloeocapsa PCC-7428* (*Gloeocapsopsis crepidinum*), *Chroococcidiopsaceae* (*Gloeocapsopsis* sp.) and *Coleofasciculaceae* (*Symplocastrum* sp.) were found to be the dominant ones, as well as *Panacagrimonas* (*P. perspica*), *Methylotenera* (*Methylophilus methylotrophus*), *Methylophilaceae* (*Methylotenera* sp.) and *Variovorax* (*V*. *guangxiensis*/*soli*). Obtained at an even greater distance from the fresco, sample 08 was dominated by communities of cyanobacteria such as *Cyanobacterales* (*Loriellopsis* sp.), *Leptolyngbyaceae* (*Myxacorys* sp., and *Stenomitos* sp.), as well as unidentified bacteria from the class *Ktedonobacteria* and the phylum *Chloroflexi*, with the sole presence of *Chloroflexaceae* (*Chloroflexus* sp.) and *Chloronema*.

#### 2.1.3. Co-Occurrence and Differential Abundance Analyses 

**Figure 5:**
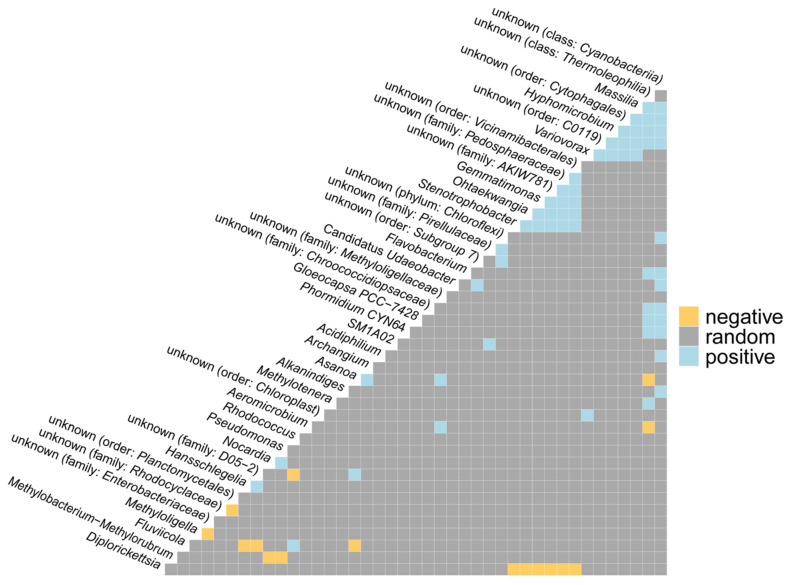
Co-occurrence analysis between detected taxa with positive, negative, and random interactions shown. Only those taxa that were present in any sample with at least 0.5% of occurrence were analyzed.

The relationships between taxonomic categories (positive, negative, and random interactions) were visualized based on the joint appearance of taxa (a co-occurrence analysis), as depicted in Figure 5. The results indicated a positive correlation between the genera *Asanoa* and *Archangium*, *Nocardia*, *Pseudomonas*, *Gemmatimonas* and *Stenotrophobacter* on the one hand and *Ohtaekwangia*, *Asanoa*, and *Acidiphilium* on the other. Moreover, *a positive correlation was observed for Flavobacterium* and *Alkanindiges* on the one hand and the unknown taxon from the class of cyanobacteria on the other. Furthermore, a positive correlation was noted between *Phormidium*, *Gloeocapsa*, and an unknown taxon from the family of *Chroococcidiopsaceae*, and unknown types from the classes of cyanobacteria and *Thermoleophilia*. A negative correlation was noted between the genera *Methylobacterium*-*Methylorubrum* and *Nocardia* on the one hand and *Pseudomonas* on the other. More negative correlations were found between *Diplorickettsia* and the genera *Stenotrophobacter*, *Ohtaekwangia* on the one hand and *Gemmatimonas* on the other, as well as between *Fluviicola* and *Hansschlegelia* on the one hand and *Alkanindiges* on the other. 

The analysis of differential abundance was used to generate a matrix of the relative abundance of statistically significant species at all the taxonomic levels based on the origin of the samples (Table 2). 

Based on the results, it was determined that only two of the known genera (*Asanoa* and *Plantactinospora*) were present to a statistically significant extent. They were found only in the samples taken from the fresco and were therefore defined as core species. For all the remaining taxa, as stated in Table 2, a statistically differential abundance was characteristic only for the samples obtained from around the fresco.

### 2.2. An Analysis of the Culturable Bacterial Communities 

**Figure 6:**
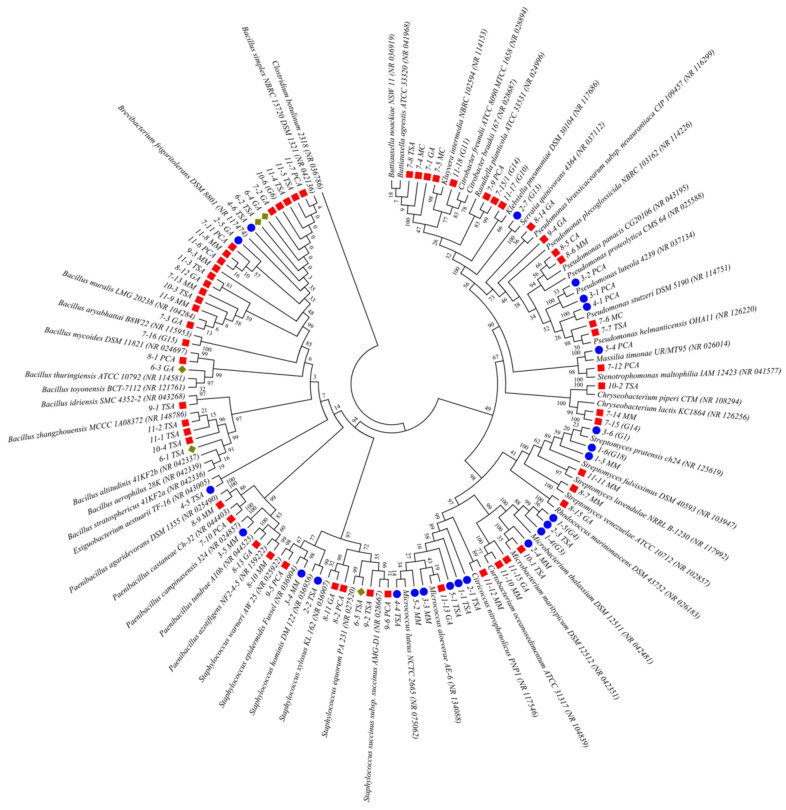
Neighbor-joining phylogenetic tree based on a pair-wise distance matrix with the Kimura two-parameter nucleotide substitution model, showing the relationship of the 16S rRNA sequences among obtained isolates from the Cave Church of Sts. Peter and Paul. The topology of the trees was evaluated by the bootstrap resampling method with 1000 replicates. *Clostridium botulinum* 2318 was used as an outgroup. (● fresco isolates; ■ around the fresco isolates; ♦ iconostasis isolates).

Following different morphology of axenic bacterial cultures, 96 potentially different colonies were obtained according to their ability for growth on various media. By analyzing the 16S rRNA sequences, ultimately, 44 different species were identified, obtained by a culturable approach (Figure 6). 

**Figure 7:**
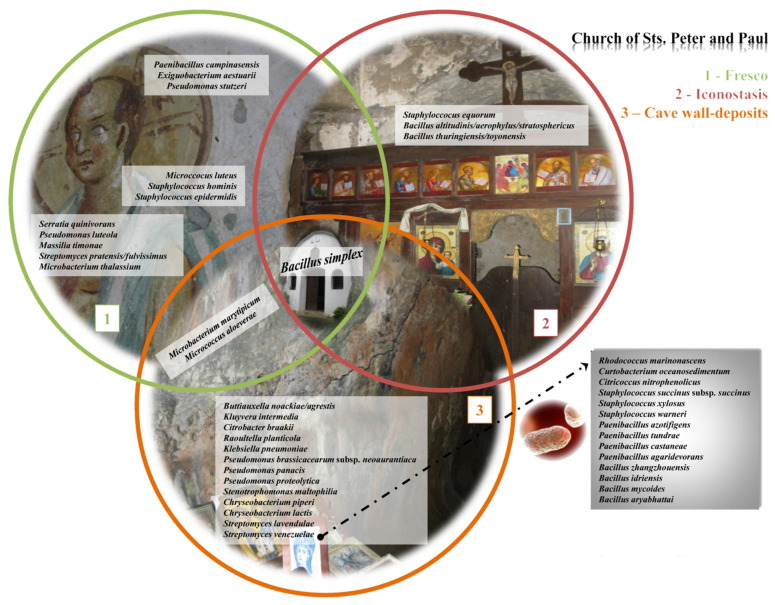
Spatial distribution of culturable bacterial isolates depending on the sampling site.

Based on the results obtained, the dominance of the genus *Bacillus* was noted in the culturable community. Furthermore, various types of the following genera were also dominant: *Paenibacillus*, *Pseudomonas*, *Microbacterium*, *Streptomyces*, *Staphylococcus*, *Rhodococcus*, *Micrococcus*, and *Curtobacterium*, while their distribution in relation to the sample location is depicted in the form of Vene’s diagram (Figure 7). The expectedly high number of unique species was characteristic of the area surrounding the fresco, while the most frequently isolated species were from the genera *Bacillus*, *Paenibacillus* and *Staphylococcus*, and *Pseudomonas*. There was a certain overlap between them and *Microbacterium marytipicum* and *Micrococcus aloeverae*, which were, at the same time, characteristic of the cave walls interior and the fresco itself, while *Bacillus simplex* was isolated from all the samples. Species characteristic and unique for the fresco itself were the following: *Serratia quinivorans*, *Pseudomonas luteola*, *Massilia timonae*, *Streptomyces pratensis/fulvissimus*, *Microbacterium thalassium*, *Micrococcus luteus*, *Staphylococcus hominis*, *Paenibacillus campinasensis*, *Exiguobacterium aestuarii*, *Pseudomonas stutzeri*, and *Staphylococcus epidermidis*.

### 2.3. Antifungal Activity

Following the initial screening of 36 bacterial antagonists against 10 biodeteriogenic fungi of the same origin (previously isolated and molecularly identified to the species level), 16 antagonists were selected, whose results are shown in Table 3. Of the 16 isolates, in a method of dual cultivation, only three isolates out of all the ones tested showed the highest percentage of growth inhibition of various fungal species at the same time (four to five species simultaneously). Based on the 16S rRNA gene they were identified as *Streptomyces fluvissimus/pratensis* (1-3 TSA), *Bacillus altitudinis/aerosphericus/stratosphericus* (6-1 TSA), and *Streptomyces lavenulae* (11-11MM). *Chryseobacterium ureilyticum* (7-15/G14) was also selected since it was one of the rare isolates (in addition to the isolate 11-11 MM) which exhibited a good antifungal effect against *P. album*. Isolate 1-3 TSA, as the best antagonist in this study, statistically significantly inhibited the growth of *P. citreonigrum*, *B. murorum*, *C. cladosporioides*, *A. aureulatus*, and *E. nigrum* at the same time. All of the aforementioned antagonists were further analyzed using genome sequencing.

### 2.4. Whole-Genome Sequencing 

The whole genomes of the best antagonists were sequenced, and the results of the statistical analysis of the genomes with the closest reference strains and characteristics are shown in Table 4.

Based on the presented statistical data, it is evident that the genomes of isolates 1-3 TSA and 11-11 MM were the largest, containing the most numerous protein-coding sequences (CDS) ranging from 7529 (1-3 TSA) and 8549 (11-11MM). However, in the case of isolate 6-1 TSA, that number was only 3923 CDS, which, at the same time, qualifies it as the smallest genome in this study, 3.8 Mb in size and with a low percentage of G+C content. The lowest G+C content was, however, noted for the 7-15/G14 isolate. Interestingly, as many as four types of Clustered Regularly Interspaced Short Palindromic Repeats (CRISPR) were noted for isolate 1-3 TSA. Based on the taxonomic annotation (a borderline ANI value of >95% indicates membership in the same type) and a phylogenetic analysis (which included a comparison with numerous protein markers) it was determined that the 1-3 TSA isolate is closest to the type strain *Streptomyces anulatus* JCM 4721 with an ANI value greater than 97%. Moreover, it was concluded that 6-1 TSA belongs to the syntype strain (each group of type strains is of equal status, on which the description and name of the new type is based) *Bacillus altitudinis* DSM 26896 (ANI 98.5%), and that 7-15/G14 belongs to the type strain *Chryseobacterium viscerum* 687B-08 (ANI > 95%). Unlike them, isolate 11-11MM was characterized only down to the genus level, as *Streptomyces* sp., since the ANI value was only confirmed to that taxonomic level and there are no similarities with any of the genomes deposited in the database (Table 5). Bearing that in mind, we can, with certainty, point out that this is a new species for science. 

The antiSMASH analysis of selected isolates showed potential for the production of different secondary metabolites. Thus, the *S. anulatus* was determined to have exceptional potential for the production (100% identical) of AmfS class III lantipeptides, isorenieratene terpenes, warkmycin cs1/cs2 polyketide angucycline, ectoine, the non-ribosomal synthesized peptide (NRP) coelichelin, the polyketide alkylresorcinol, NRP+polyketide complex sgr ptms, phenazine cluster compounds [5-Acetyl-5,10-dihydrophenazine-1-carboxylic acid; 5-(2-hydroxyacetyl)-5,10-dihydrophenazine-1-carboxylic acid; endophenazine a1/f/g], as well as siderophoric desferrioxamine b with a somewhat lower level of similarity (80%). However, the aforementioned isolate was also noted to produce melanin. Isolate 11-11 MM also had AmfS lantipeptides in common with 1-3 TSA, ectoine, and coelichelin, as well as the antibiotic azomycin, with an 83% overlap. In the case of the *B. altitudinis*, the presence of the gene for the antimicrobial peptides bacilysin, and lichenysin (for both 85%), as well as bacillibactin and fengycin was noted with a probability lower than 53%. Of the offered metabolites for *C. viscerum*, only the polyketide flexirubin had an overlap probability of 75%.

## 3. Discussion

Biodeterioration is conditioned by numerous factors such as the chemical makeup and nature of the material, exposure of the object itself, and its environment. At the same time, the frequency of cleaning and maintenance also significantly affects the further destruction of objects of cultural heritage. The cave microbiota, and especially stone colonizers, display exceptional biodiversity and complexity of types. It was previously determined that such cave ecosystems are dominated by the bacterial phyla *Proteobacteria*, *Firmicutes*, *Bacteroidetes*, and *Actinobacteria* [30], which was also confirmed in this paper. Even though certain studies indicated lower percentages of *Actinobacteria* [31], others showed the opposite—that they are in fact a dominant component in the colonization of stone surfaces. This includes the genus *Pseudonocardia* [32], which was also confirmed by the results of this study. *Thioalkalivibrio*, which belongs to the phylum *Proteobacteria* and is linked to the oxidation of sulfur, was dominant in the studied samples. A similar claim had previously also been proven [33]. In addition, *Sphingomonas*, which was obtained from the damaged parts of the wall painting [34], confirmed its dominance in our study and might be potentially a core taxa. *Actinomycetospora*, another representative of the dominant bacterial environment around the fresco and responsible for the formation of white deposits on the stone wall in our study, is believed to support the development of microbes on such a substrate [35]. Along with *Rubrobacter* and *Nocardioides*, it leads to the emergence of pink deposits on murals and wall paintings [36], which is yet again confirmed by the results of our study. *Streptomyces* species, which caused changes to the colors on the wall paintings in Egyptian tombs [37], are thought to have caused damage to the fresco itself. Even though their presence was noted to a significant extent in our study, some of them were still chosen for further study of antibiodeteriogenic activity.

In addition to real bacterial taxa, the metagenome data from our study also indicated the dominance of cyanobacterial communities, especially in samples of green and black wall patinas and deposits. The metagenome analysis confirmed the presence of many cyanobacterial strains including *Symplocastrum* sp. from the family *Coleofasciculaceae*. Until recently, this genus of cyanobacteria was insufficiently studied and lacked a molecular definition, but lately, it was proven to colonize stone surfaces and rocks, as well as lakeshores and the surfaces of freshwater sponges in the Baikal lake [38]. The *Timaviella*, which was the most abundant in one of the samples of the cave walls in our study, is a recently described genus characteristic of a cave ecosystem, and the representatives of this genus *(Timaviella circinata and Timaviella karstica*) have previously been isolated from the Giant Cave lampenflora in Italy under typical cave conditions, characterized by high humidity and constant temperature [39]. Since this newly described genus consists of few more species recently described (*T. obliquedivisa*, *T. radians*, *Timaviella* sp. WMT-WP7-NPA, and *T. edaphica* comb. nov. [40,41], their main function and ecology are still unknown. The cave environment which we studied can be considered a limiting factor due to the weak intake of nutrients, but for organisms such as cyanobacteria, it is believed to be a suitable environment for colonization and growth, whereby plaque and stains are formed on substrates [42]. Even though in caves, or on stones, it is possible to find green algae and diatoms whose numbers correlate with a higher level of humidity in such ecosystems [43], epilithic habitats are still abundant in cyanobacterial species, which represent the first phototrophic colonizers, while various epilithic and endolithic taxa also occur [44]. The environmental conditions in caves, such as slight fluctuations in the temperature, relatively constant humidity, as well as the low intensity of light (at the entrance to the cave),, or artificial lighting, represent a suitable habitat for the development of cyanobacteria. *Cyanobacteria* were often considered pioneer colonizers in numerous harsh environments, primarily due to their ability to produce exopolymer substances which enabled them to adhere to a stone surface and very quickly develop communities [45]. Numerous cyanobacteria have gelatinous capsules which play a role in the adhesion to the substrate and serve as water retainers, which further enables them to survive in hostile environments [46]. The results of our study, like the results of numerous others, indicate the dominance of aerofit cyanobacteria as the most important components of cave photosynthetic microflora, with the addition that they are predominantly found in more illuminated parts of the cave. It is necessary to mention that this cave church as a research object is affected by visitors and artificial lights, all of which have the potential to negatively impact the ecosystem and, thus, the distribution of communities in the space. By forming green-black stains, cyanobacteria contribute to the more intense weathering of rock surfaces in environments with varying humidity levels, and thanks to chromatic adaptation, they are able to adapt to various, often poor intensities of light, which are characteristic of caves. In addition, many species create discolorations which can bring about more intense destruction of wall paintings [3]. On the other hand, certain pigments, such as phycoerythrin, could lead to color transferring onto the substrates and the creation of stains [47]. Furthermore, studies such as those conducted by Albertano et al. [48] indicated that the genus *Leptolyngbya* can bind significant amounts of calcium from a basic substrate such as the material underlying a painting or fresco, but this potential is still being studied. This genus often produces biofilms in cave habitats and in humid locations [49]. The endolithic filamentous cyanobacterium *Phormidium* was found growing under black sulfate crusts which developed on limestone in Spain [50]. Some *Chroococcus* spp. possess the ability to grow and actively penetrate the stone; that is, they show potential for endolithic growth, at the same time creating tunnels and inflicting damage, but are still important biodeteriogens of stone [51]. Overall, it is well known that the activities of cyanobacteria on frescoes or murals are not limited solely to the surface, but instead to the substrate itself (the colors and deeper structures), whereby they can destroy the objects themselves by secreting organic acids. In addition, they can excrete considerable amounts of sugar and amino acids, which encourage the development of deteriogenic bacteria and fungi, whose activities in turn can contribute to the destruction of frescoes. 

Following the isolation and identification of bacterial isolates, the presence of several species of different genera was confirmed, such as *Bacillus*, *Micrococcus*, *Streptomyces*, *Staphylococcus*, *Pseudomonas*, *Chryseobacterium*, *Paenibacillus*, and *Curtobacterium.* Many of these species had previously been found on similar artifacts [52], which provided additional support for the results obtained. Most of the isolates belong to the genus *Bacillus*, the most dominant group of isolates, which is a very heterogeneous genus and includes hundreds of strains [53]. Its cosmopolitan diversity and ability to form endospores enable it to survive in unfavorable environments such as pictures, murals, and frescoes [54]. However, its sporulation can lead to an overestimation of its numbers when compared to the actual ones found on wall paintings [34]. Furthermore, the presence of the genus *Bacillus* on the wooden iconostasis can be explained by the presence of extracellular enzymes such as cellulase or lactamase-like enzymes which dissolve lignin [55]. The presence of *Staphylococcus* spp. indicates a highly likely anthropogenic or animal origin, which is also confirmed by the *S. hominis* and *S. warneri* isolates in our study, with the possibility of horizontal transfer of antibiotic resistance genes to other species [56]. In addition to its high resistance to antibiotics, it was proven that the *S. epidermidis* also plays a role in biomineralization [57]. *Curtobacterium oceanosedimentum*, another isolate in our study, has the ability to inhibit the growth of *S. epidermidis* [58]. Yet, another isolate, the opportunistic human pathogen *Massilia timonae*, also has antifungal potential since it produces chitinases, as previously indicated [59]. The genus *Paenibacillus* is already known to possess the ability to form endospores, and to produce a broad spectrum of bioactive units and volatile organic compounds [60,61], which, with several different identified species, was one of the more numerous genera in our study. In addition, its role in the bioconservation process was almost proven due to the considerable reduction in the proliferation of the mycotoxigenic fungi, which led to a reduction in the production of mycotoxin [62]. It is well known that the genus *Streptomyces* destroys material by producing bio-pigments in conditions of high salinity, that is, disruptions in the natural environment. The pigments it produces are melanins, with variations in color ranging from brown to olive green, then carotenoids of red, purple, and pink color, and actinorhodin with a blue tint. These biopigments can paint colonized surfaces and are especially resilient to various external environmental conditions [63]. *Micrococcus* is a genus with an exceptional ability to survive under stress and can also produce important metabolites and antimicrobial compounds. Previous studies described it as a genus with strong colonizing abilities on frescoes, able to damage both the glue and binding [64]. It was proven that *M. luteus* coexists with types of the genus *Streptomyces* due to *M. luteus* resilience to exoenzymes that are secreted by streptomycetes [65]. On the other hand, it was proven that individual types of microorganisms are highly effective when it comes to the biocleaning of wall paintings, as in the case of *P. stutzeri*. Its efficiency was proven in the removal of salt crusts which are the end result of the accumulation of nitrate salts on the surface of wall paintings, and which are otherwise difficult to remove using standard methods of restoration [66]. This type of biocleaning, as a biotechnological approach, is non-toxic, non-invasive, and ecologically sound. Another example of the biocleaning of wall paintings was illustrated in an example from Rome. Even though it is characterized as a decidedly human pathogen, *S. maltophilia* was proven to be very effective in removing brown deposits of protein [67]. *Stenotrophomonas maltophilia*, and the genus *Rhodococcus*, were both identified as heterotrophic bacterial communities which colonize speleothems or calcium carbonate cave deposits. Furthermore, it was determined that *S. maltophilia* can convert nitrates into nitrites and ammonia and that its calcium carbonate precipitation can contribute to the formation of cave speleothems [68]. In numerous attempts to determine whether mineralization in caves is a biogenic process or not, various studies finally confirmed this fact by isolating bacterial strains with the ability to deposit calcium carbonate. One of them was *Klebsiella pneumoniae*, which does so by using urease. It is believed that this species can successfully be used in the construction industry to repair ruptures in concrete by producing calcite crystals, as well as for the potential production of bioconcrete [69]. The genera *Buttiauxella* and *Kluyvera* were primarily isolated from unpolluted soil and water but were also found in abundance in the intestines of slugs and other mollusks [70]. Thus, their presence in a cave ecosystem is not surprising. 

In our study, four candidates were selected based on their strong antifungal activities for whole-genome sequencing. *Streptomyces anulatus* 1-3 TSA was characterized as a strain that is potentially a prime candidate for the biocontrol of biodeteriogenic fungi. This species is already known for its production of secondary metabolites which are used in the pharmaceutical industry, agrochemical industry, and agriculture [71], while its role in the induction of systemic resistance in plants and its strong antifungal effects on *Botrytis cinerea* have been indicated [72]. The aforementioned study mentioned the use of antifungal metabolites biosynthesized by *S. anulatus* S37, including nigericin, which has the greatest antifungal effect, as well as piericidin A1 and streptochlorine, which have a somewhat lower effectiveness. In addition, this species is also known for the production of antibiotics and actinomycin D [73]. What is interesting is that based on the obtained phylogeny, the isolate *B. altitudinis* 6-1 TSA was most similar to an isolate originating from the Yerba mate plant from Argentine, which has a pronounced potential for the promotion of plant growth [74]. The ability to reduce the infectivity of the oomycete *Phytophthora sojae* was proven for similar isolates by means of the production of reactive oxygen species and callose deposition on soybeans, as well as an increase in the expression of genes that react to salicylate [75]. In addition, the strong antifungal activity of *B. altitudinis* was demonstrated against *Fusarium verticillioides*, *Corynespora cassiicola*, *Fusarium oxysporum* f.sp. *pisi* and *Sclerotinia sclerotiorum* [76]. Even though the *C. viscerum* was initially isolated from dead or infected fish [77], recent studies have shown that it can be found in nature as a free-living species, as a biocontrol agent against plant pathogens with the potential to promote plant growth [78]. 

The surface of the fresco of Jesus the Infant, as well as the walls of the cave church interior, possesses an exceptional biodiversity of the microbiota. Some represent biodeteriogens, while others can successfully be used as a means of biocleaning, for removing altered and unwanted organic substances and other microorganisms from historical wall paintings and frescoes. A multidisciplinary approach is needed in the work of scientists, conservationists, and administrative bodies, with synchronized work protocols, for a step forward to be made in the field of cultural heritage protection. Over the past decades, bacterial biomineralization has been proposed as an ecologically acceptable technology for stone protection, and it is important to focus attention on that aspect as well [79]. The most important factors for the prevention of biogenic damage to the studied object include climate control, frequent cleaning, and phenomenological monitoring. Before beginning any conservation treatment, it would be necessary to prepare a detailed plan for the protection of the entire object from potential destruction, water infiltration, and the release of moisture from the soil, and only then commence with the protection of the fresco itself.

## 4. Materials and Methods

### 4.1. Sample Location

The Cave Church of the Sts. Peter and Paul, with a unique fresco painting, is located in the village of Rsovci (43°10′32″ N 22°46′35″ E), in the rocky massif of the Kalik hill, 22 km from Pirot, as we described previously [19]. The sampling procedure for all the analyses was divided into the collection of samples from the fresco itself and from the surrounding area—around the fresco (collection date: August 2020). The sample location included four points on the painted fresco depiction of Jesus (sample 01); the entire fresco except for the depiction of Jesus (sample 02); gray discoloration on the fresco (sample 03); fresco surface damage (samples 04–05)]. The following six samples were additionally obtained from the cave interior area surrounding the fresco: wooden iconostasis (sample 06), blue-green deposits on the stone wall under the fresco (sample 07), green patina on the stone wall (sample 08), black deposits on the stone wall (sample 09), white deposits on the stone wall (sample 10), and pink deposits on the stone wall (sample 11). 

### 4.2. Metabarcoding Analysis

#### 4.2.1. DNA Extraction, Library Preparation, and NGS Sequencing

Sterile Puritan™ HydraFlock™ swabs (Puritan, Guilford, ME, USA) were taken from all sampling points and stored in a DNA/RNA shield (Zymo Research, Irvine, CA, USA) during transport. The DNA extraction was performed using the Zymo BIOMICS DNA Mini Kit (Zymo Research, USA) from a minimum of two swabs collected from each sampling point, according to the manufacturer’s protocol. The concentration of isolated DNA was measured using a Qubit Fluorometric Quantitation device (Qubit 4 Fluorometer, Invitrogen, USA). Amplicon sequencing was performed using a 2 × 300 bp paired-end sequencer on a MiSeq sequencer according to the manufacturer’s instructions (Illumina, USA) at a commercial sequencing service (FISABIO, Valencia, Spain). To identify bacterial communities, the V3-V4 region of the 16S rRNA gene was amplified with defined forward (5′-CCTAGCGGGNGGCWGCAG-3′) and reverse (5′-GACTACHVGGGTATCTAATCC-3′) primers [80].

#### 4.2.2. NGS Sequencing Data Processing and Taxonomic Annotation

Sequence quality assessment was performed using the DADA2 R package [81]. Primer trimming was performed with Bbduk [82] using the same primers as during sequencing, according to the specified functions: ktrim = l (trimming at the left end), hdist = 1 (Hamming distance), copyundefined (cloning of the reference sequence of primers to represent every possibility of a degenerate base), mm = f (ignoring the middle base of a kmer), and k = 15 (k-measure length 15). All sequences that had more than three for forward and two for the reverse strand expected errors (calculated as the sum(10^(−Q/10)), where Q is the quality score) were excluded (argument: maxEE = c(3,2)), as well as sequences that were shorter than 50 bp. Sequence merger was performed with a minimum overlap of 15 bases without mismatches. Chimeric sequences were removed using default parameters in the DaDa2 R package, and all sequences shorter than 400 bp and longer than 430 bp were also eliminated. Contaminated sequences were removed using the R package “decontam” [83] and the “combined” method, which combines frequency and prevalence probabilities by using Fisher’s method to identify contaminants. The prevalence method identifies contaminants by increased prevalence in negative controls. Taxonomic annotation of 16S rRNA sequences was carried out using the SILVA138 database (https://www.arb-silva.de/documentation/release-138/, accessed on 21 December 2021). RDP Naïve Bayesian Classifier [84] with default options as implemented in the DADA2 package was used to classify taxonomy up to the genus level. The homology of the identified ASVs (Amplicon Sequence Variant) was annotated based on the BLAST best hit in the NCBI nucleotide database and the first Top10 hits with the closest species taxonomy were used for analysis. 

#### 4.2.3. Bioinformatic and Statistical Analyses

Alpha diversity was estimated using the Phyloseq R program (McMurdie & Holmes, 2013) at all taxonomic levels up to ASV. Alpha diversity indices in terms of species richness ACE, Chao1, and a number of observed species (OBS) were used. Diversity is shown through the Shannon and Gini–Simpson indices. The obtained values for the alpha indices, supported by the Kruskal–Wallis test by ranks, were used for testing significance among the samples obtained from fresco (samples 01–05) and from the cave interior (hereinafter: around fresco, samples 06–11). Statistical significance applied in all tests was *P* < 0.05. Statistical analysis was performed using the software program IBM SPSS Statistics v.23 (IBM SPSS Inc., Armonk, NY, USA). 

In the beginning, the diversity between different samples (beta diversity) was estimated at the ASV level (previously rarefied according to the sample with the lowest read count) and determined using Double Principle Coordinate Analysis—DPCoA [85]. Sequence alignment was performed using the DECIPHER::AlignSeqs with default arguments [86]. This alignment was used to create a neighbor joining tree via phangorn package 2.5.5 [87]. However, a variance stabilizing transformation is implemented in the DESeq2 package instead of rarefaction, and the multidimensional scaling transformation (MDS) was performed using Euclidean distances [88,89]. The values on the axes were scaled based on the eigenvalues (square root of the eigenvalue ratio). To visualize the distances among samples in network analysis, weighted UniFrac distances were used, which take into account the phylogenetic distances between the observed taxa in addition to the number of taxa during the calculation [90]. Before calculating distances, samples were rarefied to even depth. The size of the node corresponds to the number of connections the node has, while the edge width corresponds to the distance between the samples (closer distances are shown by thicker lines). Nodes that were not connected to other nodes are not shown, as well as connections with distances greater than 0.1 (in the case of phylum was 0.07). 

The relationships among taxonomic categories were visualized based on co-occurrence using the cooccur package [91]. Only those taxa in any sample with at least 0.5% occurrence were considered. Before running the co-occurrence analysis, all counts that were represented by less than five reads were not considered for further analysis. Only statistically significant (*P* < 0.05) co-occurrence patterns were shown. Differential abundance analysis was estimated using the DESeq2 package [88]. Starting at the genus level, the metacoder package [92] was used to generate a relative abundance matrix at all taxa levels. The matrix was filtered by removing taxa that had fewer than five read counts in at least three samples. DESeq was performed using the Wald-test statistic, log2-fold change threshold of 2, while Benjamini and Hochberg method [93] was used for p-adjustment (P-adjusted values < 0.05 were considered significant). The reduction in log2-fold changes was performed using the apeglm method [94].

### 4.3. Culturable Bacteriobiota

The collection of samples for the cultivation of bacteria was carried out on all sampling points using dry sterile swabs by dragging and rotating the swab tip onto the designated surface with present deterioration symptoms, whilst applying firm pressure to break through any biological growth present. After sampling, swabs were put on ice and transported to the laboratory under sterile conditions. In total, 750 µL of phosphate buffer (1 × PBS) was added to the swabs tubes, vortexed for a few minutes, and 100 µL of the suspension obtained from each sample was spread onto defined nutrient media (Luria-Bertani Agar (LA), Tryptic Soy Agar (TSA), Glucose Agar (GA), Minimal Medium (MM), and Cetrimide Agar (CA)), and growth was monitored for a minimum of 48 h, at 30 °C. Individual bacterial isolates to pure axenic cultures were obtained by multiple streeking onto media according to their growth requirements, and the final selection of isolates was obtained using LA medium.

Total genomic DNA was isolated by the protocol described in Dimkić et al. [95]. Molecular identification of the isolates was performed by amplification of the 16S rRNA gene with fD1Funi-16S (5′-AGAGTTTGATCCTGGCTCAG-3′) and Rp2Runi-16SR—(5′-ACGGCTACCTTGTTAGGACTT-3′) primers [96]. The PCR reaction mixture with a total volume of 25 µL per sample contained FastGene Taq 2x Ready Mix (12.5 µL, Nippon Genetics, Germany), 0.5 µL of each primer, and 10.5 µL of PCR water and 1 µL of DNA sample. The PCR reaction was performed according to the following program: initial denaturation at 95 °C for 5 min, followed by 30 cycles of denaturation at 95 °C for 40 s, primer hybridization at 54 °C for 40 s, and elongation at 72 °C for the duration of 90 s. Final elongation was performed at 72 °C for 7 min. Amplicons were purified with a purification kit (Zymo Research PCR Purification Kit, USA) and sent to a commercial service for LightRun Sanger sequencing (Eurofins Scientific, Vienna). Purified amplicons were sequenced with 907R-16S primer (5′-CCGTCAATTCMTTTRAGTTT-3′). The sequences thus obtained were searched for homology with previously sequenced genes in the GenBank database, using the National Center for Biotechnology Information’s BLAST search program for rRNA databases. To secure taxonomic relevance, the most closely related sequences of referent strains were used for phylogenetic analyses. All sequences were aligned using ClustalW multiple sequence alignment implemented in BioEdit free program ver. 7.0.5.3, and phylogenetic trees were constructed in MEGA X using the neighbor-joining method based on a pair-wise distance matrix with the Kimura two-parameter nucleotide substitution model. 

### 4.4. Whole-Genome Sequencing

For the whole-genome sequencing (WGS), four bacterial antagonists (1-3 TSA, 11-11MM, 7-15/G14, and 6-1 TSA) were selected whose DNA (previously isolated from overnight axenic cultures and purified by commercial kit—Zymo BIOMICS DNA Mini Kit (Zymo Research, USA)) was sent for commercial sequencing (Novogene Co, Cambridge Science Park, UK). A DNA library was prepared by fragmenting genomic DNA to a size of 350 bp, and the selected fragments were then polished, dA-tailed, and ligated with a full-length NEBNext adapter. The required fragments were PCR enriched by P5 and indexed P7 oligos. The Agilent^®^ 2100 bioanalyzer was used to assess the insert size. Quantitative real-time PCR (qPCR) was performed to detect the effective concentration of each library. Illumina PE150 technology was applied for sample sequencing on a NovaSeq 6000 sequencer according to the manufacturer’s instructions. 

Sequence filtering and adapter sequence trimming were started from untrimmed sequences using the trim galore 0.6.6, and additionally, 10 bp from the left side and 2–4 bp from the right side of the reads were removed, as well. Genome assembly was performed using the SPAdes genome assembler v3.15.2 program [97], using an optimal maximum k-mer of 99 using the isolate flag without SPAdes read error correction. Genome annotation was performed using the Dfast web server [98] with default options. Busco 5.2.2 was performed using the taxonomically closest ortholog database to obtain high completeness and low duplication in assembled genomes [99]. PFAM annotation was performed using the Pfam 34 database (http://ftp.ebi.ac.uk/pub/databases/Pfam/releases/Pfam34.0/, accessed on 1 October 2021) and hmmer 3.3.2 options [100]. The secondary metabolite production search was performed using the online version of the bacterial antiSMASH 6.0.1 [101] program (https://antismash.secondarymetabolites.org/#!/start, accessed on 7 October 2021). GO annotation was performed using the GO FEAT server via the following link http://computationalbiology.ufpa.br/gofeat, accessed on 7 October 2021 [102]. A multilocus phylogeny was constructed using phylophlan 3.0.2. package [103]. For each of the isolates, a different number of protein markers were used in the phylogeny [1-3 TSA and 11-11MM (6718), 7-15/G14 (3937), 6-1 TSA (2530)]. ANI (Average Nucleotide Identity) estimation was performed using fastANI 1.32 [104] with default parameters. ANI is a similarity index between a given pair of genomes that can be applied to prokaryotic organisms independently of their G+C content, and a cutoff score of >95% indicates that they belong to the same species [105].

### 4.5. In vitro Determination of Antifungal Activity

The initial antifungal activity in vitro of selected bacterial isolates from the risk 1 group (36 isolates) was tested against 10 potentially biodeteriogenic fungi of the same origin (*Penicillium citreonigrum*, *Botryotrichum murorum*, *Cladosporium cladosporioides*, *Aspergillus aureulatus*, *Epicoccum nigrum*, *Parengyodontium album*, *Botrytis cinerea*, *Beaueria pseudobassiana*, *Mortierella alpine*, and *Trichoderma viridescens*), previously isolated and molecularly identified [29]. In the initial screening, four bacterial isolates were plated simultaneously on potato dextrose agar (PDA), in the form of squares, and incubated for 24 h, at 30 ºC. The next day, the mycelial plugs of a seven-day-old fungal isolate of interest were plated in the center of the same Petri dish, and additionally incubated for the next 7 days, at 25 ºC [106]. After the initial screening, a method of dual cultivation was applied among the individually selected antagonist and the corresponding fungus at a distance of 30 mm from the mycelial plug. The controls consisted of cultures of the studied biodeteriogenic fungi without the presence of bacterial isolates. The experiment was repeated twice, with three replications for each fungus and each antagonistic isolate. The antagonistic effect of each individually tested bacterial isolate represented the difference in fungal growth compared to the control, and the Percentage of Growth Inhibition (PGI) was calculated using the following formula [107]: (PGI)% = (KR − R1)/KR × 100, where KR represents the distance (measured in mm) from the point of inoculation to the colony margin on the control dishes, and R1 is the distance of fungal growth from the point of inoculation to the colony margin on the treated dishes in the direction of the antagonist, i.e., bacterial isolates. The obtained PGI% values, after checking for normality with the Kolmogorov–Smirnov test, were processed by standard variance analysis (One-way ANOVA test). The mean separation of percentages of mycelial growth inhibition in vitro was accomplished by Tukey’s HSD (honestly significant difference) test. Statistical significance applied in all tests was *P* < 0.05. Statistical analysis was conducted by the general procedures of IBM SPSS Statistics v.23 (SPSS, Inc., Armonk, NY, USA).

## 5. Conclusions

Complete diversity of the bacterial communities of the Cave Church of Sts. Peter and Paul was obtained via non-culturable and culturable analyses and bioconservation potential of best antagonistic bacterial isolates was assessed on the basis of which it was concluded:

- The highest abundance of species was documented on the side walls of the church, while the lowest abundance was noted on the iconostasis.
- Samples from the fresco are mutually similar, as are the samples obtained from the side walls of the church.
- *Actinobacteriota* and *Proteobacteria* are dominant phyla in the majority of studied samples.
- The dominant bacterial taxa belonged to families *Methylococcaceae*, *Blastocatellaceae*, and *Nocardioidaceae* of *Thermomicrobiales* class.
- The most prevalent genera linked to the various kinds of wall deposits were *Blastocatella* and *Crossiella*.
- The number of positive correlations between bacterial genera far outnumbered the number of negative correlations.
- A total of 44 bacteria were identified, out of 96 obtained isolates, with the dominance of species from genus *Bacillus*. *Bacillus simplex* was the only isolated species simultaneously present in all investigated substrata within the church.
- The most promising antagonistic bacteria, with the potential to suppress growth of deteriogenic fungi and which represent excellent candidates for developing biocontrol strategies, are *Streptomyces anulatus* (1-3 TSA), *Bacillus altitudinis* (6-1 TSA), *Chryseobacterium viscerum* (7-15/G14), and *Streptomyces* sp. (11-11MM).
- *Streptomyces* sp. (11-11MM) represents a new species for science.
- Further research is necessary to design the most appropriate biocontrol formulation, taking into account the environment and sensitivity of the substrate, so that the desired effect of sustainable and long-term elimination of biodeteriogenic fungi can be achieved after in situ application.

## Figures and Tables

**Table 1:** Alpha diversity within the analyzed samples from fresco and around it (average values) and presented at the phylum, family, genus, and ASV taxa levels.

Indices	Description	Mean	St. Dev.	Min.	Max.	Test Statistic	Kruskal–Wallis Sign. (*P* < 0.05)
**Phylum**							
OBS	Fresco	19.0	0.8	18.0	20.0	2.927	0.087
	Around Fresco	25.3	4.5	17.0	30.0		
Chao1	Fresco	20.0	1.8	18.0	22.0	2.909	0.088
	Around Fresco	25.4	4.5	17.0	30.0		
ACE	Fresco	21.4	2.2	19.1	24.1	2.909	0.088
	Around Fresco	25.8	4.7	17.0	30.3		
Shannon	Fresco	1.5	0.1	1.4	1.7	0.567	0.451
	Around Fresco	1.7	0.3	1.2	2.0		
Gini–Simpson	Fresco	0.7	0.0	0.7	0.7	0.214	0.643
	Around Fresco	0.7	0.1	0.6	0.8		
**Family**							
OBS	Fresco	146.75	10.14	137.00	161.00	2.909	0.088
	Around Fresco	204.50	63.89	92.00	259.00		
Chao1	Fresco	154.33	10.29	146.20	169.30	2.909	0.088
	Around Fresco	210.35	68.07	92.70	280.00		
ACE	Fresco	153.05	10.34	142.60	167.30	2.909	0.088
	Around Fresco	211.05	67.50	93.50	277.60		
Shannon	Fresco	3.10	0.22	2.90	3.40	2.283	0.131
	Around Fresco	3.52	0.61	2.40	3.90		
Gini–Simpson	Fresco	0.90	0.00	0.90	0.90	2.000	0.157
	Around Fresco	0.95	0.08	0.80	1.00		
**Genus**							
OBS	Fresco	223.25	8.66	215.00	233.00	2.909	0.088
	Around Fresco	321.50	109.80	136.00	428.00		
Chao1	Fresco	230.40	11.83	220.00	242.00	2.909	0.088
	Around Fresco	329.28	113.77	138.10	442.00		
ACE	Fresco	231.18	10.15	221.90	240.40	2.909	0.088
	Around Fresco	329.55	113.73	137.90	444.60		
Shannon	Fresco	3.58	0.22	3.40	3.90	2.297	0.130
	Around Fresco	3.98	0.61	2.90	4.40		
Gini–Simpson	Fresco	0.93	0.05	0.90	1.00	1.500	0.221
	Around Fresco	0.97	0.05	0.90	1.00		
**ASV**							
OBS	Fresco	735.00	47.63	689.00	790.00	0.727	0.394
	Around Fresco	1209.17	616.55	254.00	1757.00		
Chao1	Fresco	755.53	49.62	710.20	814.60	0.727	0.394
	Around Fresco	1253.83	644.91	254.30	1847.10		
ACE	Fresco	752.38	47.74	712.50	808.10	0.727	0.394
	Around Fresco	1246.60	638.87	254.70	1822.80		
Shannon	Fresco	5.00	0.24	4.70	5.30	0.732	0.392
	Around Fresco	5.23	1.04	3.40	6.30		
Gini–Simpson	Fresco	1.00	0.00	1.00	1.00	0.667	0.414
	Around Fresco	0.98	0.04	0.90	1.00		

**Table 2:** Analysis of the differential representation of statistically significant species according to the origin of the samples.

Taxa	Taxa Rank	Fresco				Around Fresco						Test Statistic			
		01	02	03	04–05	06	07	08	09	10	11	lfcSE	stat	P_value_	P_adj_
*Solimonadaceae*	Family	6	19	0	65	4	10111	2852	3491	1121	509	1.376	3.669	0.0002	0.0048
*Chroococcidiopsaceae*		5	0	0	0	0	4994	870	887	14	0	2.191	3.664	0.0002	0.0048
*Hyphomicrobiaceae*		6	0	4	6	0	1701	3133	1378	2502	463	1.357	5.051	0.0000	0.0000
*Rhodocyclaceae*		0	0	0	0	0	951	0	12	779	0	2.604	8.569	0.0000	0.0000
*Intrasporangiaceae*		0	0	4	0	9	66	30	376	62	53	1.431	3.579	0.0003	0.0057
*Oxalobacteraceae*		0	0	4	3	0	1377	49	877	280	414	1.511	4.125	0.0000	0.0011
*Microcystaceae*		0	0	0	0	0	33	437	56	0	333	2.157	3.608	0.0003	0.0056
*Chthonomonadaceae*		0	2	0	0	0	44	329	38	31	27	1.769	3.120	0.0018	0.0194
*env.OPS 17*		0	0	0	0	0	334	321	317	17	423	1.768	4.774	0.0000	0.0001
*Cellvibrionaceae*		0	0	0	0	3	101	40	15	71	27	1.511	4.069	0.0000	0.0011
*B1-7BS*		14	34	41	75	0	0	0	0	0	0	1.721	**−2.977**	0.0029	0.0288
*Acidobacteriae*		0	0	0	0	0	56	149	154	26	291	1.724	4.287	0.0000	0.0006
*Saprospiraceae*		0	0	0	0	0	79	146	79	15	0	2.106	3.048	0.0023	0.0236
*Micavibrionaceae*		0	0	0	0	0	4	23	138	7	19	1.940	2.950	0.0032	0.0308
*Fimbriimonadaceae*		0	0	0	0	6	22	29	6	14	20	1.610	3.260	0.0011	0.0142
*Phormidium CYN64*	Genus	0	0	0	0	0	21581	3008	79	0	0	2.834	9.142	0.0000	0.0000
*Panacagrimonas*		6	19	0	65	4	9544	2565	3201	1089	509	1.371	3.612	0.0003	0.0056
*Gloeocapsa PCC-7428*		0	0	0	0	0	2921	198	156	8	0	2.354	9.806	0.0000	0.0000
*Hyphomicrobium*		6	0	4	6	0	1692	3075	1352	2496	303	1.374	4.961	0.0000	0.0000
*Asanoa*		1260	509	605	43	0	0	0	0	0	17	1.723	**−3.203**	0.0014	0.0166
*Rhodococcus*		16	5	0	6	20	180	12	2212	601	31	1.398	3.329	0.0009	0.0127
*Afipia*		0	0	0	0	0	370	52	21	352	0	2.159	3.521	0.0004	0.0066
*Knoellia*		0	0	0	0	0	66	0	342	62	34	2.132	3.315	0.0009	0.0130
*Massilia*		0	0	0	0	0	1195	36	864	150	348	1.834	5.058	0.0000	0.0000
*Chalicogloea CCALA 975*		0	0	0	0	0	33	422	56	0	333	2.154	3.599	0.0003	0.0056
*Chthonomonas*		0	2	0	0	0	44	329	38	31	27	1.769	3.120	0.0018	0.0194
*Plantactinospora*		183	75	121	0	0	0	0	0	0	0	2.054	**−3.015**	0.0026	0.0259
*Cellvibrio*		0	0	0	0	3	101	40	15	71	27	1.511	4.069	0.0000	0.0011
*Pseudofulvimonas*		0	0	0	0	0	11	47	150	34	48	1.769	3.555	0.0004	0.0060
*Ferruginibacter*		0	0	0	0	0	36	59	197	0	65	2.089	3.139	0.0017	0.0194
*Aureimonas*		0	0	0	0	17	109	37	102	41	0	1.841	3.761	0.0002	0.0035
*Paludibaculum*		0	0	0	0	0	56	149	154	26	291	1.724	4.287	0.0000	0.0006
*Rhodoferax*		0	0	0	0	5	80	14	138	0	0	2.218	2.799	0.0051	0.0447
*Leptothrix*		0	0	0	0	0	109	196	153	23	21	1.769	3.995	0.0001	0.0015
*Pedobacter*		0	0	0	0	0	139	7	20	105	0	2.157	2.799	0.0051	0.0447
*Rhodopseudomonas*		0	0	0	0	21	29	11	0	81	0	2.259	2.878	0.0040	0.0381
*P3OB-42*		0	0	0	0	0	45	59	72	6	23	1.773	3.248	0.0012	0.0145
*Sandaracinus*		0	0	0	0	5	58	18	36	4	25	1.599	3.482	0.0005	0.0075
*Roseisolibacter*		0	0	0	0	0	7	120	179	0	64	2.152	3.090	0.0020	0.0210

To adjust the probability (P_adjusted values_ < 0.05 were considered significant), the Benjamini–Hochberg method was used. The bold values represent core species only taken from the fresco samples.

**Table 3:** Antifungal activity in vitro of selected antagonistic bacterial isolates against biodeteriogenic fungi.

	*Penicillium*	*Botryotrichum*	*Cladosporium*	*Aspergillus*	*Epicoccum*	*Parengyodontium*	*Botrytis*	*Beaueria*	*Mortierella*	*Trichoderma*
1-2 TSA	0.0 ± 0.00 ^c^	2.0 ± 1.96 ^e^	46.1 ± 5.52 ^b^	18.2 ± 5.25 ^cde^	0.0 ± 0.00 ^e^	0.0 ± 0.00 ^d^	0.0 ± 0.00 ^e^	1.7 ± 1.67 ^e^	0.0 ± 0.00 ^e^	0.0 ± 0.00 ^b^
1-3 TSA	**70.2 ± 4.91 ^a^**	**70.6 ± 5.66 ^a^**	**80.9 ± 2.55 ^a^**	**51.5 ± 0.00 ^a^**	**66.0 ± 4.91 ^a^**	0.0 ± 0.00 ^d^	0.0 ± 0.00 ^e^	27.5 ± 1.44 ^c^	0.0 ± 0.00 ^e^	0.0 ± 0.00 ^b^
6-1 TSA	**78.7 ± 0.00 ^a^**	0.0 ± 0.00 ^e^	0.0 ± 0.00 ^d^	0.0 ± 0.00 ^e^	31.9 ± 2.46 ^bc^	16.1 ± 0.55 ^bc^	50.5 ± 0.00 ^b^	**62.5 ± 4.33 ^a^**	**42.0 ± 0.00 ^a^**	**79.0 ± 0.58 ^a^**
7-3 GA	19.1 ± 2.46 ^b^	13.7 ± 4.53 ^de^	52.9 ± 6.79 ^b^	0.0 ± 0.00 ^e^	0.0 ± 0.00 ^e^	13.9 ± 4.81 ^c^	0.0 ± 0.00 ^e^	0.0 ± 0.00 ^e^	10.1 ± 1.67 ^d^	0.0 ± 0.00 ^b^
7-6 MC	29.8 ± 6.14 ^b^	37.3 ± 0.00 ^b^	19.1 ± 7.64 ^cd^	30.3 ± 5.25 ^bc^	0.0 ± 0.00 ^e^	11.1 ± 3.21 ^c^	0.0 ± 0.00 ^e^	0.0 ± 0.00 ^e^	0.0 ± 0.00 ^e^	0.0 ± 0.00 ^b^
7-10 PCA	0.0 ± 0.00 ^c^	45.1 ± 2.26 ^b^	33.8 ± 4.25 ^bc^	39.4 ± 7.00 ^ab^	0.0 ± 0.00 ^e^	24.8 ± 1.46 ^ab^	0.0 ± 0.00 ^e^	0.0 ± 0.00 ^e^	0.0 ± 0.00 ^e^	0.0 ± 0.00 ^b^
7-15/G14	0.0 ± 0.00 ^c^	11.8 ± 1.13 ^de^	55.9 ± 0.00 ^ab^	18.2 ± 5.25 ^cde^	0.0 ± 0.00 ^e^	**27.6 ± 3.21 ^a^**	0.0 ± 0.00 ^e^	0.0 ± 0.00 ^e^	0.0 ± 0.00 ^e^	0.0 ± 0.00 ^b^
7-16/G15	0.0 ± 0.00 ^c^	33.3 ± 2.26 ^bc^	44.1 ± 5.10 ^bc^	39.4 ± 3.50 ^ab^	0.0 ± 0.00 ^e^	0.0 ± 0.00 ^d^	0.0 ± 0.00 ^e^	15.0 ± 0.00 ^d^	0.0 ± 0.00 ^e^	0.0 ± 0.00 ^b^
8-5 CA	0.0 ± 0.00 ^c^	19.6 ± 7.92 ^cd^	55.9 ± 1.70 ^ab^	21.2 ± 3.50 ^bcd^	0.0 ± 0.00 ^e^	0.0 ± 0.00 ^d^	0.0 ± 0.00 ^e^	17.5 ± 1.44 ^cd^	**37.7 ± 0.84 ^a^**	0.0 ± 0.00 ^b^
8-7 MM	26.3 ± 7.48 ^b^	0.0 ± 0.00 ^e^	0.0 ± 0.00 ^d^	0.0 ± 0.00 ^e^	25.5 ± 1.23 ^c^	0.0 ± 0.00 ^d^	46.2 ± 1.24 ^c^	15.0 ± 2.89 ^d^	0.0 ± 0.00 ^e^	0.0 ± 0.00 ^b^
8-10 MM	0.0 ± 0.00 ^c^	15.7 ± 1.13 ^de^	42.6 ± 7.64 ^bc^	24.2 ± 1.75 ^bcd^	0.0 ± 0.00 ^e^	0.0 ± 0.00 ^d^	0.0 ± 0.00 ^e^	7.5 ± 4.33 ^de^	0.0 ± 0.00 ^e^	0.0 ± 0.00 ^b^
8-14 GA	0.0 ± 0.00 ^c^	47.1 ± 3.39 ^b^	38.2 ± 8.49 ^bc^	36.4 ± 1.75 ^abc^	0.0 ± 0.00 ^e^	0.0 ± 0.00 ^d^	0.0 ± 0.00 ^e^	15.0 ± 2.89 ^d^	21.7 ± 0.00 ^c^	0.0 ± 0.00 ^b^
9-2 TSA	0.0 ± 0.00 ^c^	0.0 ± 0.00 ^e^	50.0 ± 8.49 ^b^	9.1 ± 0.00 ^de^	0.0 ± 0.00 ^e^	0.0 ± 0.00 ^d^	0.0 ± 0.00 ^e^	7.5 ± 1.44 ^de^	29.0 ± 2.51 ^b^	0.0 ± 0.00 ^b^
10-4 TSA	0.0 ± 0.00 ^c^	0.0 ± 0.00 ^e^	0.0 ± 0.00 ^d^	24.2 ± 1.75 ^bcd^	0.0 ± 0.00 ^e^	16.7 ± 0.00 ^bc^	0.0 ± 0.00 ^e^	40.0 ± 2.89 ^b^	20.3 ± 0.84 ^c^	0.0 ± 0.00 ^b^
11-10 MM	0.0 ± 0.00 ^c^	39.2 ± 1.13 ^b^	0.0 ± 0.00 ^d^	18.2 ± 5.25 ^cde^	36.2 ± 2.46 ^b^	16.7 ± 0.00 ^bc^	35.5 ± 2.48 ^d^	1.7 ± 1.67 e	30.4 ± 3.35 ^ab^	0.0 ± 0.00 ^b^
11-11 MM	0.0 ± 0.00 ^c^	37.3 ± 2.27 ^b^	0.0 ± 0.00 ^d^	18.2 ± 1.75 ^cde^	**61.7 ± 2.46 ^a^**	**27.6 ± 3.21 ^a^**	**63.4 ± 1.24 ^a^**	10.0 ± 2.89 ^de^	**36.2 ± 0.00 ^a^**	0.0 ± 0.00 ^b^

Values followed by the same letter per column are not statistically significant according to Tukey’s HSD test. The most statistically significant values of PGI (%) are shown in bold. Tested fungal species: *Penicillium citreonigrum*, *Botryotrichum murorum*, *Cladosporium cladosporioides*, *Aspergillus aureulatus*, *Epicoccum nigrum*, *Parengyodontium album*, *Botrytis cinerea*, *Beaueria pseudobassiana*, *Mortierella alpine*, and *Trichoderma viridescens.*

**Table 4:** Genome annotation and comparison with the closest reference strains.

	1-3 TSA	6-1 TSA	7-15/G14	11-11MM
Total Sequence Length (bp):	8,497,050	3,834,536	5,315,248	9,378,728
Number of Sequences:	53	52	38	161
Longest Sequences (bp):	1,277,654	677,502	1,550,329	615,461
N50 (bp):	373,280	420,792	1,252,813	151,700
Gap Ratio (%):	0.015652	0.005216	0.005832	0.00853
GCcontent (%):	71.6	41.2	36.2	71.8
Number of CDSs:	7529	3923	4672	8549
Average Protein Length:	332.3	286.6	327.9	322.4
Coding Ratio (%):	88.3	88	86.5	88.2
Number of rRNAs:	2	3	3	4
Number of tRNAs:	85	75	80	94
Number of CRISPRs:	4	0	0	2
**Assembly according to the closest reference strain**				
# contigs (≥0 bp)	71	78	45	
# contigs (≥1000 bp)	38	24	16	
# contigs (≥5000 bp)	33	22	11	
# contigs (≥10,000 bp)	33	22	10	
# contigs (≥25,000 bp)	32	19	9	
# contigs (≥50,000 bp)	28	15	9	
Total length (≥0 bp)	8,499,567	3,838,568	5,316,373	
Total length (≥1000 bp)	8,492,054	3,824,321	5,308,269	
Total length (≥5000 bp)	8,483,321	3,818,604	5,297,689	
Total length (≥10,000 bp)	8,483,321	3,818,604	5,292,390	
Total length (≥25,000 bp)	8,464,247	3,769,813	5,281,078	
Total length (≥50,000 bp)	8,322,500	3,615,438	5,281,078	
# contigs	38	24	16	
Largest contig	1,277,654	677,502	1,550,329	
Total length	8,492,054	3824321	5,308,269	
Reference length	8,855,698	3,812,514	5,693,782	
GC (%)	71.61	41.24	36.15	
Reference GC (%)	71.71	41.11	36.16	
N50	373,280	420,792	1,252,813	
NG50	373,280	420,792	773,259	
N75	237,595	268,237	598,143	
NG75	237,278	268,237	407,868	
L50	7	4	2	
LG50	7	4	3	
L75	14	7	4	
LG75	15	7	5	
# misassemblies	251	71	149	
# misassembled contigs	29	14	8	
Misassembled contigs length	8,168,390	3,470,420	5,209,481	
# local misassemblies	570	69	329	
# scaffold gap ext. mis.	5	0	1	
# scaffold gap loc. mis.	59	0	2	
# unaligned mis. contigs	2	1	1	
# unaligned contigs	1 + 35 part	2 + 14 part	5 + 10 part	
Unaligned length	1,427,995	306,517	1,225,761	
Genome fraction (%)	79.357	92.168	71.617	
Duplication ratio	1.005	1.001	1.001	
# N’s per 100 kbp	15.66	5.23	5.84	
# mismatches per 100 kbp	2167.9	1198.66	3504.82	
# indels per 100 kbp	111.29	24.9	85.42	
# genomic features	5912 + 606 part	3670 + 107 part	3986 + 304 part	
Largest alignment	208,439	269,167	139,195	
Total aligned length	7,060,676	3,515,775	4,081,960	
NA50	38,599	86510	30,553	
NGA50	36,902	86510	27,840	
NA75	11,869	39,117	6088	
NGA75	8663	39,911	-	
LA50	63	13	45	
LGA50	68	13	52	
LA75	154	30	126	
LGA75	180	29	-	

**Table 5:** Taxonomic annotation and phylogenetic analysis of selected isolates.

Species Name	Strain No.	Accession No.	NCBI Taxonomy IDs	Type Strain	Validated	ANI (%)	Folded Fragments	Total Fragments
**1-3 TSA**								
*Streptomyces anulatus*	strain=JCM 4721	GCA_014650675.1	1892	type	TRUE	97.0620	2441	2811
*Streptomyces anulatus*	strain=ATCC 11523	GCA_001434355.1	1892	syntype	TRUE	97.0122	2443	2811
*Streptomyces griseus*	strain=NCTC13033	GCA_900460065.1	1911	type	TRUE	93.1318	2171	2811
*Streptomyces griseus*	strain=DSM 40236	GCA_900105705.1	1911	syntype	TRUE	93.1183	2214	2811
*Streptomyces anulatus*	strain=NRRL B-3362	GCA_000717105.1	1892	syntype	TRUE	92.8788	1974	2811
*Streptomyces anulatus*	strain=NRRL B-2873	GCA_000721175.1	1892	syntype	TRUE	92.8387	1967	2811
*Streptomyces griseus*	strain=JCM 4516	GCA_014655335.1	1911	syntype	FALSE	92.5514	2195	2811
**6-1 TSA**								
*Bacillus altitudinis*	strain=DSM 26896	GCA_000949525.1	293387	syntype	TRUE	98.4941	1160	1263
*Bacillus altitudinis*	strain=NIO-1130	GCA_900094985.1	293387	syntype	TRUE	97.9919	1150	1263
*Bacillus altitudinis*	strain=NIO-1130	GCA_001457015.1	293387	type	TRUE	97.9919	1150	1263
*Bacillus altitudinis*	strain=41KF2b	GCA_000691145.1	293387	type	TRUE	97.9481	1154	1263
*Bacillus xiamenensis*	strain=HYC-10	GCA_000300535.1	1178537	type	TRUE	91.4340	1029	1263
*Bacillus zhangzhouensis*	strain=DW5-4	GCA_000715205.1	1178540	type	TRUE	89.9647	1085	1263
**7-15/G14**								
*Chryseobacterium viscerum*	strain=687B-08	GCA_002899945.2	1037377	type	TRUE	95.4107	1497	1760
*Chryseobacterium sediminis*	strain=IMT-174	GCA_008386505.1	1679494	type	TRUE	87.2802	1354	1760
*Chryseobacterium rhizoplanae*	strain=DSM 29371	GCA_900182655.1	1609531	type	TRUE	87.2214	1367	1760
*Chryseobacterium cucumeris*	strain=GSE06	GCA_001593385.1	1813611	type	TRUE	86.4695	1357	1760
*Chryseobacterium candidae*	strain=JC507	GCA_004916905.1	1978493	type	TRUE	86.3433	1199	1760
*Chryseobacterium gleum*	strain=ATCC 35910	GCA_000143785.1	250	type	TRUE	85.8473	1296	1760
*Chryseobacterium aureum*	strain=17S1E7	GCA_003971235.1	2497456	type	TRUE	85.8461	1326	1760
*Chryseobacterium gleum*	strain=NCTC11432	GCA_900636535.1	250	type	TRUE	85.8060	1312	1760
*Chryseobacterium gleum*	strain=FDAARGOS_1103	GCA_016766875.1	250	type	TRUE	85.7937	1318	1760
*Chryseobacterium culicis*	strain=DSM 23031	GCA_900108365.1	680127	type	TRUE	85.5231	1323	1760
*Chryseobacterium jejuense*	strain=DSM 19299	GCA_900100075.1	445960	type	TRUE	83.2892	1147	1760
*Chryseobacterium ureilyticum*	strain=DSM 18017	GCA_900156735.1	373668	type	TRUE	83.1204	1126	1760
*Chryseobacterium nakagawai*	strain=NCTC13529	GCA_900637665.1	1241982	type	TRUE	82.9748	1147	1760
**11-11 MM**								
*Streptomyces vinaceus*	strain=JCM 4849	GCA_014656155.1	1960	type	TRUE	86.7781	1870	3057
*Streptomyces xanthophaeus*	strain=NBRC 12829	GCA_016755895.1	67385	type	TRUE	86.7459	1974	3057
*Streptomyces xanthophaeus*	strain=NRRL B-5414	GCA_000725805.1	67385	type	TRUE	86.7181	1950	3057
*Streptomyces goshikiensis*	strain=JCM 4640	GCA_014650555.1	1942	type	TRUE	86.6907	1786	3057
*Streptomyces nojiriensis*	strain=JCM 3382	GCA_014648615.1	66374	type	TRUE	86.6852	1933	3057
*Streptomyces spororaveus*	strain=NBRC 15456	GCA_016755875.1	284039	type	TRUE	86.6749	1886	3057
*Streptomyces nojiriensis*	strain=NBRC 13794	GCA_016755855.1	66374	type	TRUE	86.6735	1924	3057
*Streptomyces avidinii*	strain=JCM 4726	GCA_014650695.1	1895	type	TRUE	86.5841	1893	3057
*Streptomyces subrutilus*	strain=ATCC 27467	GCA_008704535.1	36818	type	TRUE	86.4921	1785	3057
*Streptomyces subrutilus*	strain=JCM 4834	GCA_014650935.1	36818	type	TRUE	86.4834	1766	3057
*Streptomyces cinnamonensis*	strain=JCM 4019	GCA_014648795.1	1900	type	TRUE	86.4664	1876	3057
*Streptomyces cinnamonensis*	strain=NBRC 15873	GCA_016755835.1	1900	type	TRUE	86.4132	1914	3057
*Streptomyces virginiae*	strain=NRRL ISP-5094	GCA_000720455.1	1961	type	TRUE	86.4089	1896	3057

## Data Availability

All data have been deposited in the NCBI database as BioProject ID: PRJNA826742 (under the accessions from SAMN27618500 to SAMN27618509).

## Supplementary Materials

The supporting information can be downloaded at: https://www.mdpi.com/article/10.3390/ijms24021016/s1.
