# Supplementary material for: Bacteriobiota of the Cave Church of Sts. Peter and Paul in Serbia—Culturable and Non-Culturable Communities’ Assessment in the Bioconservation Potential of a Peculiar Fresco Painting
Source: Int J Mol Sci. 2023 Jan 5;24(2):1016. doi: 10.3390/ijms24021016 (PMC9867463; doi:10.3390/ijms24021016)
Supplement: Supplementary file 1 [file ijms-24-01016-s001.zip › ijms-2096534-supplementary.pdf]

SUPPLEMENTARY MATERIAL

Article

Bacteriobiota of the Cave Church of Sts. Peter and Paul in Serbia – culturable and non-culturable communities' assessment in the bioconservation potential of a peculiar fresco painting

Ivica Dimkić [1], Milica Ćopić [1], Marija Petrović [1], Miloš Stupar [1], Željko Savković [1], Aleksandar Knežević [1], Gordana Subakov Simić [1], Milica Ljaljević Grbić [1], and Nikola Unković [1,*]

[1] University of Belgrade – Faculty of Biology, Studentski trg 16, 11 000 Belgrade, Serbia
*Correspondence: unkovicn@bio.bg.ac.rs; Tel.: +381 11 3244 847

Table S1: Number of the read retention and sequence decontamination.

Sample	Input	Primer remove	Quality filter	Denoised F	Denoised R	Merged	Chimera remove	Length trim	Decontaminated reads	Percent retained	Final % of reads
01	223434	223273	168088	166348	166780	161680	157149	157081	156829	99.84	70.30
02	154666	154512	116104	114690	115138	111196	108664	108562	108332	99.79	70.19
03	182100	181849	134976	133872	134152	130475	126900	126861	126159	99.45	69.67
04-05	167557	167294	125791	123930	124634	120416	117894	117732	117545	99.84	70.26
06	94529	84983	61253	59735	59777	54033	53659	50099	50099	100	53.00
07	191108	190877	148381	145616	146235	140016	137667	137566	137345	99.84	71.98
08	170400	169778	133325	129521	130810	123097	120704	120378	119975	99.67	70.64
09	177903	177494	138534	135773	136545	129765	124052	123820	123426	99.68	69.60
10	176302	176146	133088	131824	132061	128694	126278	125828	125784	99.97	71.37
11	199758	199377	153129	148751	150247	140028	133902	133493	133464	99.98	66.83
Control	367	350	193	156	170	156	156	156	-	-	42.51

Table S2: Alpha diversity within the analyzed individual samples from fresco painting and from sampling points of the cave interior and presented at the phylum, family, genus, and ASV level.

Sample	Description	OBS	Chao1	SE Chao1	ACE	SE ACE	Shannon	Gini-Simpson	Inv Simpson	level
01	Depiction of Jesus	689	710.2	7.9	712.5	13.2	4.7	1.0	43.6	ASV
02	Entire fresco except depiction of Jesus	702	719.1	8.0	712.7	12.9	5.0	1.0	46.7	
03	Gray discoloration on fresco	759	778.2	7.9	776.2	13.5	5.0	1.0	39.8	
04-05	Fresco surface damage	790	814.6	9.6	808.1	13.8	5.3	1.0	81.0	
06	Iconostasis	254	254.3	0.7	254.7	7.8	3.4	0.9	8.2	
07	Blue-green deposits on stone wall	1311	1380.0	16.1	1366.6	18.0	5.2	1.0	32.3	
08	Green patina on stone wall	1757	1847.1	18.7	1822.8	20.8	5.9	1.0	85.8	
09	Black deposits on stone wall	1522	1560.0	10.4	1556.8	19.4	5.8	1.0	95.4	
10	White deposits on stone wall	667	684.7	7.2	685.2	12.9	4.8	1.0	54.9	
11	Pink deposits on stone wall	1744	1796.9	12.7	1793.5	20.8	6.3	1.0	212.7	
01	Depiction of Jesus	228	242.0	8.1	239.5	7.5	3.5	0.9	12.8	Genus
02	Entire fresco except depiction of Jesus	217	220.4	3.0	221.9	7.0	3.5	0.9	11.7	
03	Gray discoloration on fresco	215	220.0	3.6	222.9	7.1	3.4	0.9	10.0	
04-05	Fresco surface damage	233	239.2	4.4	240.4	7.3	3.9	1.0	21.9	
06	Iconostasis	136	138.1	2.5	137.9	5.7	2.9	0.9	7.2	
07	Blue-green deposits on stone wall	428	442.0	6.5	444.6	10.3	4.2	1.0	24.8	
08	Green patina on stone wall	401	407.0	4.2	406.9	9.5	4.4	1.0	34.3	
09	Black deposits on stone wall	371	383.5	6.8	382.8	9.2	4.4	1.0	40.4	
10	White deposits on stone wall	249	252.3	2.6	255.5	7.7	3.6	0.9	14.4	
11	Pink deposits on stone wall	344	352.8	6.0	349.6	8.7	4.4	1.0	36.7	
01	Depiction of Jesus	145	152.3	5.7	152.0	5.9	3.0	0.9	8.6	Family
02	Entire fresco except depiction of Jesus	137	146.2	7.4	142.6	5.4	3.1	0.9	8.0	
03	Gray discoloration on fresco	144	149.5	4.3	150.3	5.7	2.9	0.9	6.7	
04-05	Fresco surface damage	161	169.3	6.4	167.3	6.1	3.4	0.9	12.5	
06	Iconostasis	92	92.7	1.1	93.5	4.7	2.4	0.8	5.2	
07	Blue-green deposits on stone wall	259	280.0	10.5	277.6	8.0	3.8	1.0	20.4	
08	Green patina on stone wall	249	252.9	3.3	253.5	7.3	3.9	1.0	24.2	
09	Black deposits on stone wall	240	243.9	3.1	246.3	7.3	3.9	1.0	25.7	
10	White deposits on stone wall	168	172.4	3.6	173.9	6.4	3.2	0.9	10.6	
11	Pink deposits on stone wall	219	220.2	1.5	221.5	6.5	3.9	1.0	27.0	

01	Depiction of Jesus	19	19.0	0.2	20.3	1.9	1.4	0.7	2.9	
02	Entire fresco except depiction of Jesus	19	22.0	4.6	22.1	2.0	1.5	0.7	3.1	
03	Gray discoloration on fresco	18	18.0	0.5	19.1	1.4	1.4	0.7	2.9	
04-05	Fresco surface damage	20	21.0	2.3	24.1	1.7	1.7	0.7	3.9	
06	Iconostasis	17	17.0	0.0	17.0	1.9	1.2	0.6	2.6	
07	Blue-green deposits on stone wall	30	30.0	0.2	30.3	2.6	1.8	0.8	4.2	**Phylum**
08	Green patina on stone wall	28	28.0	0.2	28.5	2.3	2.0	0.8	5.7	
09	Black deposits on stone wall	27	27.0	0.2	27.6	2.3	1.7	0.7	3.5	
10	White deposits on stone wall	24	24.3	0.9	25.4	1.9	1.3	0.6	2.6	
11	Pink deposits on stone wall	26	26.0	0.0	26.0	2.0	2.0	0.8	5.6	

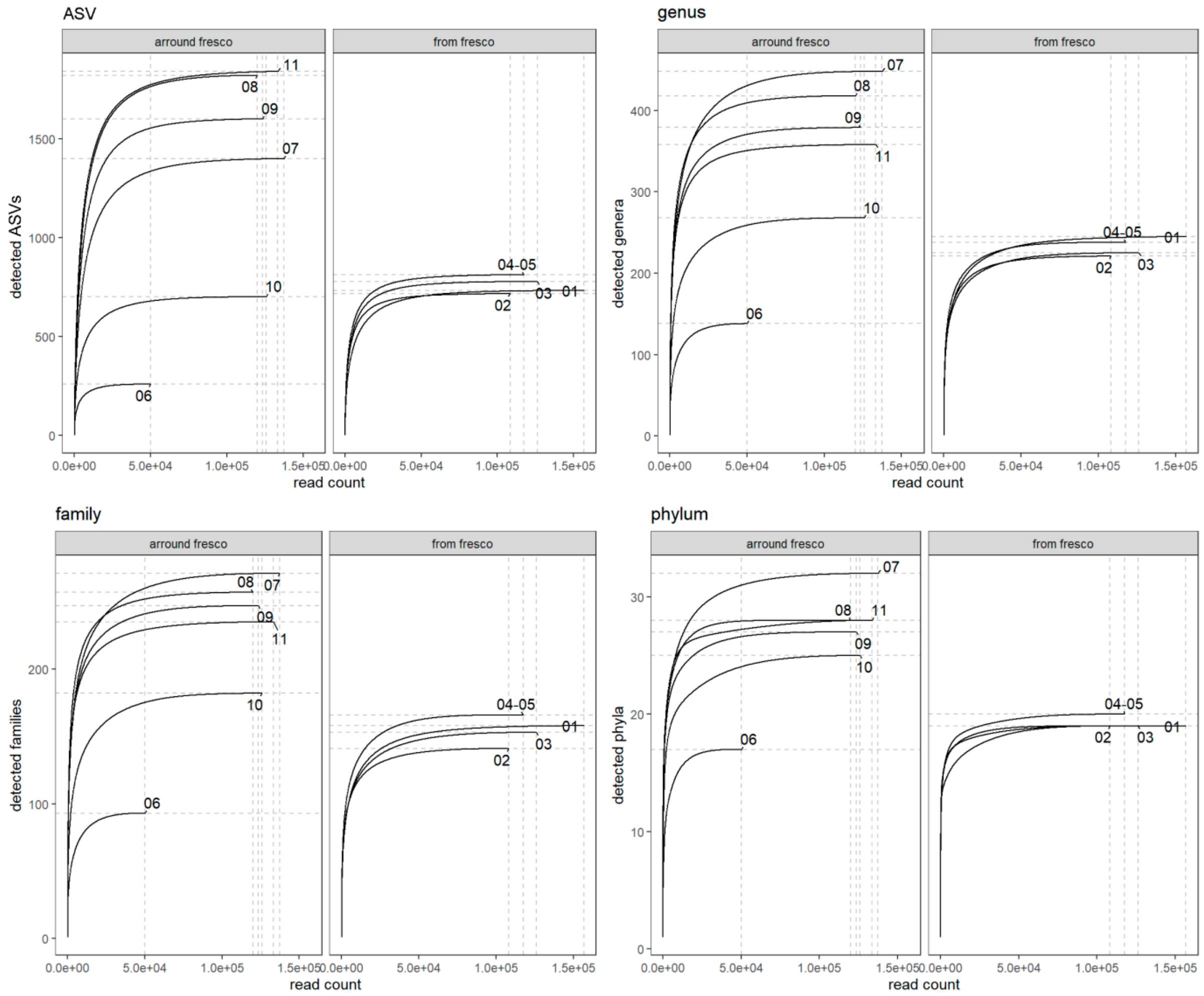

Figure S1: Rarefaction analysis at the ASV, genus, family and phylum levels.

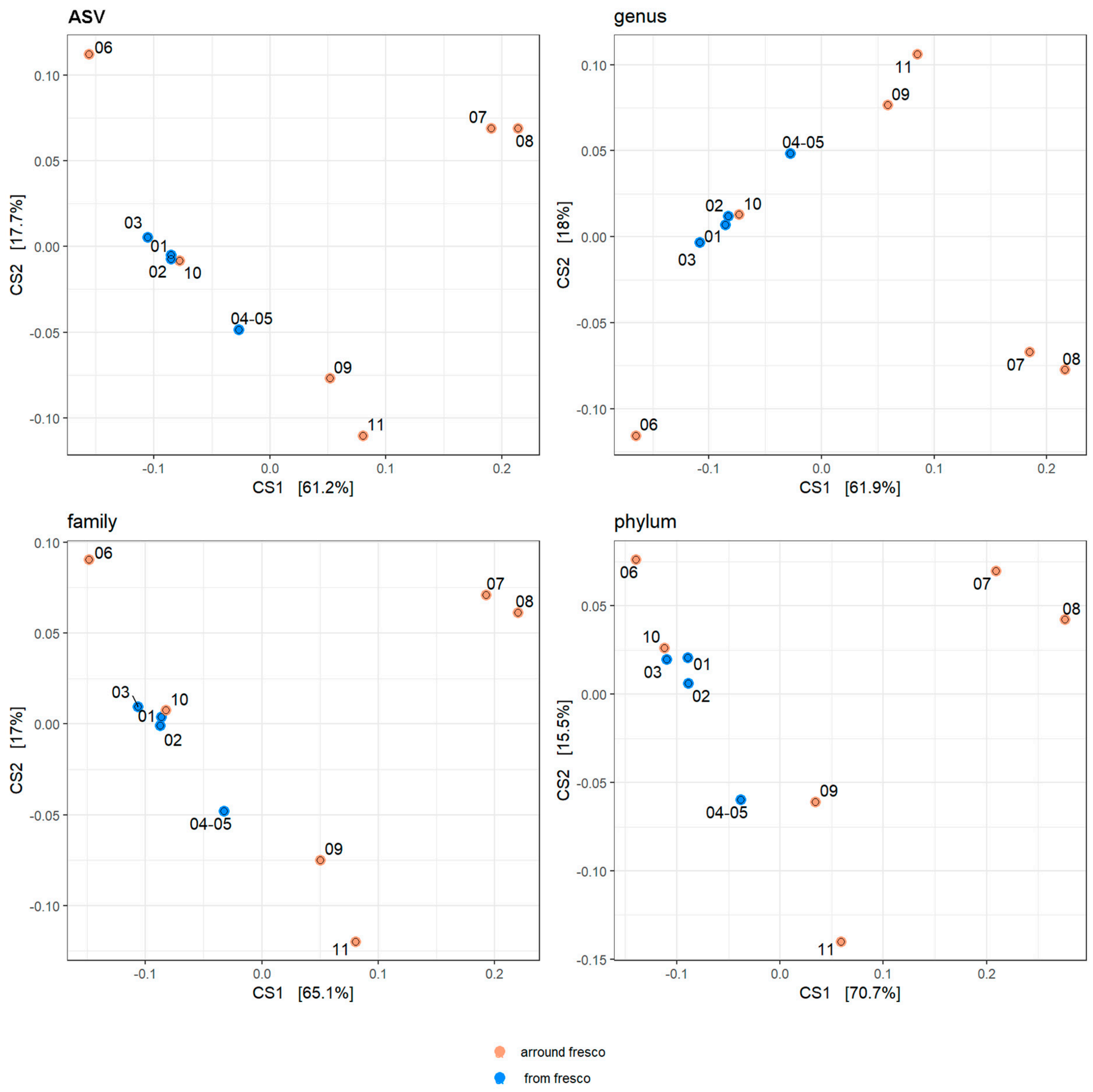

Figure S2: The DPCoA plot created with Bray-Curtis dissimilarities at the ASV, genus, family, and phylum taxa levels, to present the variability within and between groups of samples.
